# Structural Reconstruction of Cu_2_O Superparticles toward Electrocatalytic CO_2_ Reduction with High C_2+_ Products Selectivity

**DOI:** 10.1002/advs.202105292

**Published:** 2022-04-01

**Authors:** Yawen Jiang, Xinyu Wang, Delong Duan, Chaohua He, Jun Ma, Wenqing Zhang, Hengjie Liu, Ran Long, Zibiao Li, Tingting Kong, Xian Jun Loh, Li Song, Enyi Ye, Yujie Xiong

**Affiliations:** ^1^ Hefei National Laboratory for Physical Sciences at the Microscale Frontiers Science Center for Planetary Exploration and Emerging Technologies School of Chemistry and Materials Science National Synchrotron Radiation Laboratory University of Science and Technology of China Hefei Anhui 230026 China; ^2^ Institute of Materials Research and Engineering A*STAR (Agency for Science, Technology and Research) 2 Fusionopolis Way, Innovis, #08‐03 Singapore 138634 Singapore; ^3^ College of Chemistry and Chemical Engineering Xi'an Shiyou University Xi'an Shaanxi 710054 China

**Keywords:** CO_2_ electroreduction, Cu_2_O superparticle, in situ spectroscopy, multicarbon products, structural reconstruction

## Abstract

Structural reconstruction is a process commonly observed for Cu‐based catalysts in electrochemical CO_2_ reduction. The Cu‐based precatalysts with structural complexity often undergo sophisticated structural reconstruction processes, which may offer opportunities for enhancing the electrosynthesis of multicarbon products (C_2+_ products) but remain largely uncertain due to various new structural features possibly arising during the processes. In this work, the Cu_2_O superparticles with an assembly structure are demonstrated to undergo complicated structure evolution under electrochemical reduction condition, enabling highly selective CO_2_‐to‐C_2+_ products conversion in electrocatalysis. As revealed by electron microscopic characterization together with in situ X‐ray absorption spectroscopy and Raman spectroscopy, the building blocks inside the superparticle fuse to generate numerous grain boundaries while those in the outer shell detach to form nanogap structures that can efficiently confine OH^−^ to induce high local pH. Such a combination of unique structural features with local reaction environment offers two important factors for facilitating C−C coupling. Consequently, the Cu_2_O superparticle‐derived catalyst achieves high faradaic efficiencies of 53.2% for C_2_H_4_ and 74.2% for C_2+_ products, surpassing the performance of geometrically simpler Cu_2_O cube‐derived catalyst and most reported Cu electrocatalysts under comparable conditions. This work provides insights for rationally designing highly selective CO_2_ reduction electrocatalysts by controlling structural reconstruction.

## Graphical abstract

It is highly desired yet challenging to achieve electrochemical CO_2_ reduction toward multicarbon products. Here, a unique “planet–satellite”‐like Cu structure is formed through structural reconstruction from Cu_2_O superparticles to demonstrate high faradaic efficiencies of 53.2% for ethylene and 74.2% for C_2+_ products in electrochemical CO_2_ reduction.

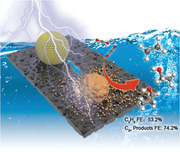

## 1 Introduction

Electrocatalytic CO_2_ reduction reaction (CO_2_RR) to produce valuable multicarbon products (C_2+_ products) is a very promising strategy for meeting the energy and environmental issues associated with continuing consumption of fossil fuels.^[^ ^1^ ^]^ Numerous electrocatalysts have been explored for CO_2_ electroreduction, among which Cu is the most promising metal that can produce C_2+_ products with appreciable yields due to its moderate binding energy to *CO intermediate.^[^ ^2^ ^]^ However, the rational design of Cu‐based catalysts is never an easy task because structural reconstruction is an inevitable phenomenon under electrochemical CO_2_ reduction potential. The structures and chemical states of the pristine catalysts (also called precatalysts) may undergo great changes by applied potentials or adsorbates, such as fragmentation, agglomeration, and reshaping.^[^ ^3^ ^]^ Such a situation may offer opportunities for enhancing the electrosynthesis of C_2+_ products. For instance, highly active stepped surfaces could be formed through the in situ electrochemical activations of Cu nanowires, showing a high faradaic efficiency (FE) toward C_2_H_4_.^[^ ^4^ ^]^ It was also demonstrated that the Cu_2_O nanoparticles on carbon black, with cysteamine acting as immobilization agent, could be transformed into small fragmented Cu nanoparticles with dense boundaries during CO_2_RR, promoting C–C coupling reaction.^[^ ^3a^ ^]^ Disordered structures can be created from Cu nanoparticles by electrochemical nanocrystal scrambling, which were intrinsically active for low overpotential C_2+_ formation.^[^ ^5^ ^]^ It is worth pointing out that the structural reconstruction also remains large uncertainty to the CO_2_RR performance. In certain cases, the Cu‐based catalysts may lose electrocatalytic activity and selectivity during the structural reconstruction. This uncertainty makes the rational design of Cu‐based catalysts toward highly selective conversion of CO_2_ to C_2+_ products still a challenging task despite the vast development in the past years. As such, the reasonable selection of Cu‐based precatalysts and the deep understanding of their structural evolution hold the key to achieve high performance for C_2+_ products generation.

**Figure 1:**
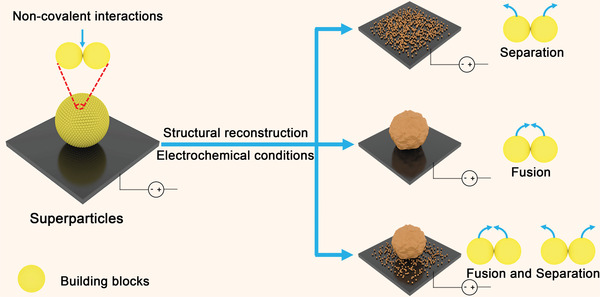
Schematic illustration of the possible structural reconstruction processes for the superparticle under electrochemical reduction conditions.

In terms of precatalysts selection, we pay special attention to the precatalysts with structural complexity as they often undergo sophisticated and unique structural reconstruction processes. In many cases, complex nanostructures show superior catalytic activity and selectivity toward target products to their geometrically simpler counterparts.^[^ ^3^, ^6^ ^]^ Colloidal superparticles, which are self‐assembled from size‐ and shape‐controlled nanoparticles, are such a class of materials with complex structures.^[^ ^7^ ^]^ Due to the unique assembly structures and coupling effects, the superparticles have shown excellent performance in photocatalysis,^[^ ^8^ ^]^ photonic device,^[^ ^9^ ^]^ and biomedical applications.^[^ ^10^ ^]^ The building blocks of the superparticle are held together by the relatively weak noncovalent interactions such as van der Waals forces and the interactions induced by the surface functionalities of nanocrystals.^[^ ^7^ ^]^ In principle, the building blocks in the superparticle may separate from each other or fuse to form larger particles under cathodic potentials, constituting complex structures (**Figure** **1**). Intuitively, when Cu‐based superparticles are selected as precatalysts for electrochemical CO_2_ reduction, fusion can induce the formation of grain boundaries while separation may produce nanogaps to confine OH^−^ species for sustaining high local pH, both of which are important factors for accelerating C–C coupling.^[^ ^2^ ^]^ To this end, the Cu‐based superparticles are the potential precatalysts to form highly active reconstructed structures for C_2+_ products generation. However, the structural evolution of superparticles under electrochemical CO_2_ reduction potential remains largely unexplored.

To prove the concept that the Cu‐based superparticles can induce the formation of highly active reconstructed structures for C_2+_ products generation, the Cu_2_O superparticle with a simple synthetic method is chosen as the model precatalyst for electrochemical CO_2_RR, as oxide‐derived Cu is an efficient class of catalysts for C_2+_ products generation. As a central theme, the structural reconstruction behavior of Cu_2_O superparticle is examined via pre‐electroreduction by a chronopotentiometry (CP) method. We reveal that during the preelectroreduction, the building blocks inside the superparticle fuse to form large aggerates with numerous grain boundaries while those in the outer shell detach to produce many nanoscale/sub‐nanoscale spacings between Cu facets which can efficiently confine OH^−^ to induce high local pH, as evidenced by electron microscopic characterization together with in situ X‐ray absorption spectroscopy (XAS) and Raman spectroscopy. Ultimately, the Cu_2_O superparticle‐derived catalyst achieves excellent faradaic efficiencies (FEs) of 53.2% for ethylene and 74.2% for C_2+_ products, significantly higher than those of the Cu catalyst derived from the widely used but geometrically simpler Cu_2_O cube and also surpassing most reported Cu‐based electrocatalysts.

## 2 Results and Discussion

### 2.1 Synthesis and Pre‐Electroreduction of Cu_2_O Precatalysts

**Figure 2:**
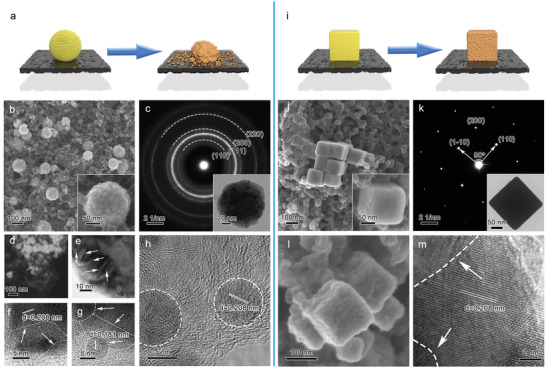
a) Schematic illustration for the structural evolution of the Cu_2_O superparticle after pre‐electroreduction. b) SEM image of the Cu_2_O superparticle on Ketjen black carbon. c) TEM image and the corresponding SAED pattern of the Cu_2_O superparticle. d) STEM image of Cu catalyst derived from Cu_2_O superparticle via CP (Cu_2_O superparticle‐CP3). e–g) TEM and HRTEM images of the large Cu aggregate in Cu_2_O superparticle‐CP3. The arrows indicate the formed grain boundaries. h) HRTEM image of the small detached Cu nanoparticles in Cu_2_O superparticle‐CP3. i) Schematic illustration for the structural evolution of the Cu_2_O cube after pre‐electroreduction. j) SEM image of the Cu_2_O cube on Ketjen black carbon. k) TEM image and the corresponding SAED pattern of the Cu_2_O cube. l) SEM and m) HRTEM images of Cu catalyst derived from Cu_2_O cube via CP (Cu_2_O cube‐CP3). The arrows indicate the formed grain boundaries.

Cu_2_O superparticles are firstly synthesized based on a reported polyvinyl pyrrolidone (PVP)‐assisted polyol method, which can be then transformed into Cu nanostructures via pre‐electroreduction (**Figure** **2**a).^[^ ^11^ ^]^ According to the previous studies, the formation process of the Cu_2_O superparticle follows the “two‐stage growth model” with polyvinyl PVP as a capping agent.^[^ ^9^ ^]^ In the first stage, nucleation takes place to produce nanosized primary Cu_2_O particles. Next, the primary Cu_2_O nanoparticles aggregate to form a larger secondary superstructure. Scanning electron microscopy (SEM) image shows that the Cu_2_O superparticle has a spherical shape with an average size of 130 nm and is composed of many smaller nanoparticles (Figure 2b;Figure S1, Supporting Information). Moreover, transmission electron microscopy (TEM) and scanning transmission electron microscopy (STEM; Figure S2, Supporting Information), taken from the Cu_2_O superparticle specimen cut by focused ion beam (FIB), clearly reveals that the Cu_2_O superparticle is assembled by small crystallites with 4–9 nm size, which matches well with the size (7.7 nm) estimated from the calculation for the strongest (111) peak in its XRD pattern using the Debye–Scherrer formula (Figure S1, Supporting Information). The building blocks, which are integrated by van der Waals forces and PVP,^[^ ^12^ ^]^ keep separated without forming grain boundaries and are randomly oriented as indicated by the selected‐area electron diffraction (SAED) pattern (Figure 2c). According to the high‐resolution TEM (HRTEM) image (Figure S3, Supporting Information), the distance between two adjacent planes in a specific direction is measured to be 0.246 nm, corresponding to the (111) planes of face‐centered cubic (fcc) Cu_2_O. Note that such Cu_2_O superparticles, covered by a thin layer of PVP (Figures S2 and S4, Supporting Information), are very stable. Even after being exfoliated by ultrasonic cell pulverizer for 12 h, the small Cu_2_O building blocks are still bound together (Figure S2, Supporting Information).

We then prepare the ink by mixing the Cu_2_O superparticle with Ketjen black carbon (a good conductive support) and Nafion solution in isopropanol, which is then dispersed on glass carbon electrode (0.196 cm^2^) as a precatalyst. Subsequently, the Cu_2_O superparticle is pre‐electroreduced by the CP method at a cathodic current density of −3 mA cm^−2^ for 1000 s in CO_2_‐saturated 0.1 M KHCO_3_ solution (Figure S5, Supporting Information), which can ensure sufficient reconstruction.^[^ ^13^ ^]^ Such a Cu catalyst formed through the pre‐electroreduction of Cu_2_O superparticle at −3 mA cm^−2^ is marked as Cu_2_O superparticle‐CP3.

The structure evolution of the Cu_2_O superparticle is directly observed by SEM (Figure S6, Supporting Information). As the in‐lens secondary electron detector cannot clearly distinguish the Cu catalyst from Ketjen black carbon, the energy selective backscattered electron detector is employed to reflect the element contrast of the sample. By this detector, we can clearly see that the superstructure of Cu_2_O superparticles is decomposed. The resulting structure is composed of large aggregates (tens to hundreds of nanometers) and many small nanoparticles. The large aggregates are closely surrounded by dense small nanoparticles (Figure S7, Supporting Information), forming abundant nanoscale or sub‐nanoscale spacings between Cu facets. This observation is consistent with STEM (Figure 2d) and energy‐dispersive X‐ray spectroscopy (EDS) element mapping (Figures S8 and S9, Supporting Information). According to HRTEM images (Figures 2e–g), the lattice fringes of the large aggregates are measured to be 0.208and 0.181 nm, which can be indexed to the (111) and (200) plane of fcc Cu, suggesting the Cu_2_O superparticle has been reduced to metallic Cu. Meanwhile, many grain boundaries can be observed on the large Cu aggregates. The small detached nanoparticles are of rounded shape and have an average diameter of 13.2 nm (Figure 2h;Figure S10, Supporting Information), whose lattice fringes also belong to metallic Cu.

Based on the above results, we can conclude that during the pre‐electroreduction process, the Cu_2_O superparticle is reduced to metallic Cu accompanied by both fusion and separation processes. The small building blocks in the interior of the Cu_2_O superparticle fuse together to generate the large Cu aggregate while those in the outer region detach to form the small Cu nanoparticles, constituting a “planet–satellite”‐like structure (Figure 2a). Such a reconstruction process leads to the formation of many grain boundaries and nanoscale/sub‐nanoscale spacings between Cu facets as we anticipate. In addition, PVP has been stripped off during pre‐electroreduction according to the Fourier transform infrared spectroscopy measurement and X‐ray photoelectron spectroscopy analysis (Figures S11 and S12, Supporting Information), manifesting that the capping agent would not have an impact on further CO_2_RR. We have also tried to reduce the Cu_2_O superparticle at different current densities (Figure S13, Supporting Information). Increasing the reduction of current density does not make obvious changes for the structure evolution. However, when the Cu_2_O superparticle is reduced at a very low current density of −0.5 mA cm^−2^ (Cu_2_O superparticle‐CP0.5), the building blocks are more favorable to fuse to form large aggregates while substantially less particles are detached.

Cu_2_O cubic nanocrystals, a widely studied but geometrically simpler catalyst in electrochemical CO_2_RR,^[^ ^3^, ^6^, ^13^, ^14^ ^]^ are prepared as a reference sample (Figure 2i–k). The Cu_2_O cube possesses a single‐crystal structure with continuous and periodic atom arrangement differently from the Cu_2_O superparticle, but has an average edge length of 129 nm, similarly to the size of the Cu_2_O superparticle (Figures 2j–k and Figure S14, Supporting Information). Although a small amount of PVP is also used in the synthesis process, less PVP remains on the surface of the prepared Cu_2_O cube (Figure S4, Supporting Information). The Cu_2_O cube also goes through pre‐electroreduction using the same method. In contrast to the Cu_2_O superparticle, the Cu_2_O cube maintains its cubic shape without collapse after pre‐electroreduction at −3 mA cm^−2^ (marked as Cu_2_O cube‐CP3, Figure S6, Supporting Information), consistent with the previous research.^[^ ^13^ ^]^ The SEM‐EDS mapping and STEM‐EDS mapping (Figure S8 and S9, Supporting Information) also confirm the remained Cu_2_O cubic shape. However, the surface of the Cu_2_O cube becomes rough and many grain boundaries are also formed (Figure 2l). According to HRTEM images (Figure 2m), the sample after reduction typically has the lattice fringe of 0.208 nm, suggesting that the Cu_2_O cube has been reduced to metallic Cu.

### 2.2 Evaluation of CO_2_ Electroreduction Performance

The electrochemical CO_2_RR is conducted using a typical H‐type cell with three electrodes in CO_2_‐saturated 0.1 m KHCO_3_ electrolyte. After the pre‐electroreduction of the Cu_2_O precatalysts at −3 mA cm^−2^, stepped‐potential electrolysis is performed between −0.85 and −1.25 V versus reversible hydrogen electrode (RHE). To benchmark the performance of our Cu_2_O superparticle‐CP3 and Cu_2_O cube‐CP3 catalyst, commercial Cu particles (Figure S15, Supporting Information) are also tested for electrocatalytic CO_2_RR. The gas products and liquid products are analyzed by gas chromatography and ^1^H nuclear magnetic resonance (NMR) spectroscopy (Figure S16, Supporting Information), respectively.

**Figure 3:**
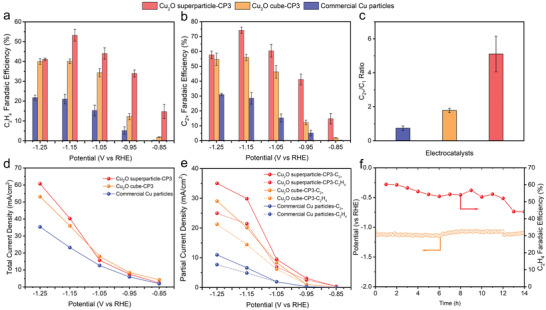
Faradaic efficiencies of a) C_2_H_4_ and b) C_2+_ products for the three types of catalysts. c) C_2+_/C_1_ faradaic efficiency ratio on the three types of catalysts at −1.15 V (vs RHE). d) Total current densities and e) partial current densities of C_2_H_4_ and C_2+_ products for the three types of catalysts at the tested potentials. The current density (normalized by geometric area) is the average value of triplicate measurements. f) Stability test of Cu_2_O superparticle‐CP3 at −40 mA cm^−2^.

As shown in **Figure** **3**a,b, Cu_2_O superparticle‐CP3 gives the highest FEs for ethylene and C_2+_ products among the three catalysts. The Cu_2_O superparticle‐CP3 produces CO as the dominant product of CO_2_RR with a FE of 26.4% at −0.85 V (vs RHE) (Figure S17, Supporting Information). As the potential is more negative, the FE of CO decreases accompanied by the gradually increased selectivities of C_2_H_4_ and other C_2+_ products, indicating that *CO undergoes dimerization. At −1.15 V (vs RHE), the Cu_2_O superparticle‐CP3 obtains the maximum FE of C_2+_ products (74.2%), with C_2_H_4_, C_2_H_5_OH, and C_3_H_7_OH accounting for 53.2%, 12.7%, and 8.3%, respectively. In contrast, the FEs of ethylene and C_2+_ products are 40.1% and 55.9% for Cu_2_O cube‐CP3, and 21% and 28.5% for commercial Cu particles, respectively. Moreover, Cu_2_O superparticle‐CP3 achieves a 2.9‐fold enhancement in the ratio of C_2+_ to C_1_ compared to Cu_2_O cube‐CP3 and a 6.9‐fold enhancement over commercial Cu particles at the optimal potential of −1.15 V (vs RHE) (Figure 3c). Note that the high FEs of C_2_H_4_ and C_2+_ products achieved by our Cu_2_O superparticle‐CP3 are also among the best when benchmarked against Cu‐based catalysts under comparable conditions in previous literature (Table S1, Supporting Information).

As another important parameter, the partial current densities of C_2_H_4_ and C_2+_ products by the Cu_2_O superparticle‐CP3 are also higher as compared with the two reference catalysts (Figure 3d,e). Moreover, the Cu_2_O superparticle‐CP3 exhibits the lowest H_2_ FE, suggesting H_2_ evolution reaction (HER) has been effectively suppressed (Figure S18, Supporting Information). Electrochemical impedance spectroscopy measurement also shows that Cu_2_O superparticle‐CP3 has the lowest charge transfer resistance (Figure S19, Supporting Information). Taken together, the results strongly indicate that Cu_2_O superparticle‐CP3 is more favorable for C–C coupling to produce C_2+_ products during electrochemical CO_2_RR. Notably, the “planet–satellite”‐like structure of Cu_2_O superparticle‐CP3 is well preserved after reaction at each fixed potential (Figure S20, Supporting Information). To assess performance durability, a long‐term constant current density electrolysis of −40 mA cm^−2^ under continuous pure CO_2_ flow is conducted. As shown in Figure 3f, Cu_2_O superparticle‐CP3 can maintain over 50% FE of C_2_H_4_ for 12 h. However, beyond 13 h, the C_2_H_4_ FE decays below 50%. STEM images (Figure S21, Supporting Information) reveal that as the electrolysis time prolongs, the number of small satellite particles decreases accompanied by the gradual increase of their size, which may cause the decrease of the C_2_H_4_ FE.

### 2.3 In Situ Characterization and Mechanism Investigation

**Figure 4:**
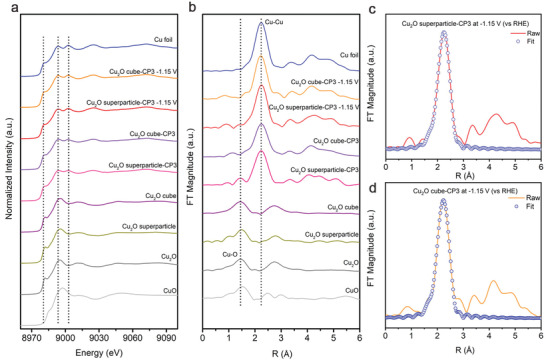
a) In situ Cu K‐edge XANES spectra of the Cu_2_O superparticle‐CP3 and Cu_2_O cube‐CP3. b) The corresponding FT‐EXAFS spectra. EXAFS fitting curves in R space of c) Cu_2_O superparticle‐CP3 and d) Cu_2_O cube‐CP3 at −1.15 V (vs RHE).

The information gleaned above has indicated that our Cu_2_O superparticle‐derived catalyst offers excellent selectivity for C_2+_ products generation. Note that some previous works stated that the Cu^+^ species in the oxide‐derived Cu catalysts is responsible for the improvement of C–C coupling.^[^ ^15^ ^]^ As the oxide‐derived Cu can be easily reoxidized upon exposure to air, we employ in situ XAS to track the real chemical and structure states of the catalysts during CO_2_RR (Figure S22, Supporting Information). As shown in the Cu K‐edge X‐ray absorption near edge structure (XANES) spectra (**Figure** **4**a) and the corresponding K^2^‐weighted Fourier‐transformed extended X‐ray absorption fine structure (FT‐EXAFS) spectra (Figure 4b), Cu_2_O superparticle‐CP3 is fully reduced to metallic Cu at −1.15 V (vs RHE, the optimal potential) during CO_2_RR. Thus, the improvement of C_2+_ products generation for Cu_2_O superparticle‐CP3 is not attributed to the Cu^+^ species.

Upon excluding the possibility of Cu^+^ species, we look into other possible factors. The electron microscopic characterizations above have revealed that Cu_2_O superparticle‐CP3 is composed of two components—large Cu aggregates and 13.2 nm Cu rounded nanoparticles. In particular, many grain boundaries are observed on the large Cu aggregates. It has been recognized that the grain boundary acts as a key structural feature to the C_2+_ products generation.^[^ ^2^ ^]^ The coordinatively unsaturated sites with strong CO binding were proposed to exist on the grain boundaries, which can increase the concentration of adsorbed CO to undergo further C–C coupling.^[^ ^16^ ^]^ Density functional theory calculations also showed that the active sites at the grain boundary plane can reduce the formation energy of *C_2_ intermediates.^[^ ^17^ ^]^ For this reason, we propose that the grain boundary on the large Cu aggregate should be one of the key factors to the high selectivity of C_2+_ products by Cu_2_O superparticle‐CP3. To support this argument, we prepare the Cu‐rounded nanoparticle with a size (14.6 nm) close to that of small satellite particles in Cu_2_O superparticle‐CP3 (13.2 nm), whose electrochemical CO_2_RR performance is assessed for comparison (Figure S23, Supporting Information). It turns out that the prepared small Cu rounded nanoparticles on Ketjen black carbon without grain boundaries exhibit a significantly higher selectivity for H_2_ (31–62% FE) and a substantially lower C_2+_ selectivity (below 42% FE) compared to Cu_2_O superparticle‐CP3. Such a finding is consistent with a previous study that the Cu‐rounded nanoparticles with sizes of 5−15 nm mainly produce H_2_ and have very limited ability for C–C coupling.^[^ ^18^ ^]^ As a result, the grain boundary should be a factor beneficial to the C–C coupling over Cu_2_O superparticle‐CP3.

To resolve the local structural information for the grain boundaries, we further perform the quantitative EXAFS curve fitting analysis for the obtained catalysts (Figure 4c,d; Figure S24, Supporting Information). The analysis indicates that the coordination number (CN) of Cu atom for Cu_2_O cube‐CP3 is 10.88 at −1.15 V (vs RHE), which is very close to that of Cu_2_O superparticle‐CP3 (CN = 10.86; Table S2, Supporting Information). This suggests that the structural reconstruction on both Cu_2_O superparticle and Cu_2_O cube can induce the formation of grain boundaries with low‐coordinated Cu atoms in similar density. However, Cu_2_O superparticle‐CP3 shows substantially higher C−C coupling ability than Cu_2_O cube‐CP3.

**Figure 5:**
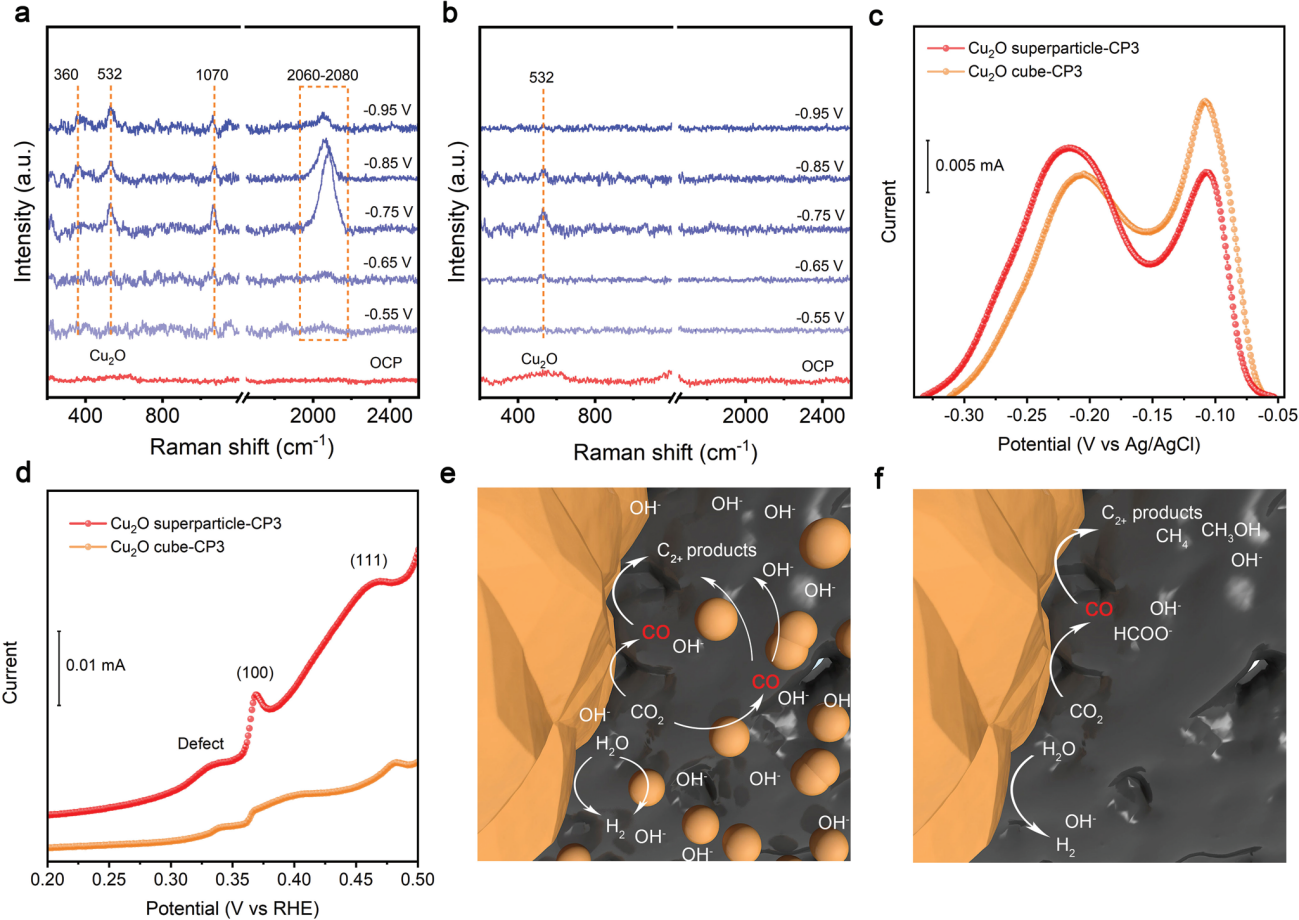
In situ Raman spectra of a) Cu_2_O superparticle‐CP3 and b) Cu_2_O cube‐CP3 during CO_2_RR. c) Underpotential deposition of lead: the Pb monolayer stripping peaks. d) Voltammograms of OH_ads_ peaks collected in 0.1 m KOH solution. Schematic illustration for the mechanism of enhanced C–C coupling of e) Cu_2_O superparticle‐CP3 compared to f) Cu_2_O cube‐CP3.

Then a question naturally arises what factor causes the different C−C coupling ability of two oxide‐derived Cu catalysts. To decode the mechanism, in situ Raman spectroscopy is further employed to characterize the reaction system (Figure S25, Supporting Information). Before pre‐electroreduction, both the Cu_2_O superparticle and Cu_2_O cube exhibit three peaks at 415, 534, and 620 cm^−1^, corresponding to the modes resulting from a multi‐phonon process^[^ ^19^ ^]^—the T_2g_ vibrational mode^[^ ^20^ ^]^ and the T_1u_ mode of Cu_2_O (Figure S26, Supporting Information).^[^ ^21^ ^]^ The peak at ≈1335 and 1600 cm^−1^ are attributed to the D and G band of Ketjen black carbon.^[^ ^22^ ^]^ We further perform the in situ Raman spectroscopy after the pre‐electroreduction of the Cu_2_O catalysts in the potential range of −0.55 to –−0.95 V (vs RHE). At more negative potentials, tremendous gas bubbles are generated on the surface of the working electrode so that the Raman signal cannot be obtained. As shown in **Figure** **5**a,b, the peaks assigned to Cu_2_O appear in the spectra of Cu_2_O superparticle‐CP3 and Cu_2_O cube‐CP3 at open circuit potential,, suggesting the reduced Cu_2_O has undergone reoxidation under ex situ condition. At −0.55 V (vs RHE), the peak of Cu_2_O disappears in both cases, further confirming that the Cu_2_O‐derived catalysts have been fully reduced to metallic Cu under cathodic potentials.

To gain more insights into CO_2_RR reaction mechanism, we further look into the in situ Raman spectra of Cu_2_O superparticle‐CP3 at various potentials. The peaks at 360, 532, and 2060–2080 cm^−1^ are assigned to the Cu–CO stretching mode, the adsorbed OH group (vibration of Cu–OH) and the C–O stretching mode of *CO on atop sites, respectively.^[^ ^23^ ^]^ In particular, the *CO peak at 2060–2080 cm^−1^ appears at −0.65 V (vs RHE) and becomes more intense at −0.75 V (vs RHE). As the potential further changes more negative to −0.95 V (vs RHE), the peak intensity of *CO decreases gradually, suggesting that *CO has been converted to C_2_H_4_ and other products. As for Cu_2_O cube‐CP3, the *CO peak is so weak that cannot be observed clearly, consistent with the electrochemical CO_2_RR result that it mainly produces HCOOH/H_2_ and cannot generate as much *CO intermediate as Cu_2_O superparticle‐CP3 does at low overpotentials (Figure S17, Supporting Information).

To collect information for catalyst−CO_2_ interaction, we pay special attention to the peak at 1070 cm^−1^ that can be assigned to the adsorbed carbonate (CO_3_^2−^).^[^ ^23^ ^]^ This peak appears obviously in the in situ Raman spectra of Cu_2_O superparticle‐CP3 but can hardly be observed in the spectra of Cu_2_O cube‐CP3. It is recognized that the surface‐enhanced Raman spectroscopy (SERS) effect depends on the Cu structures, which may cause the difference in the peak intensity of the adsorbed CO_3_^2−^.^[^ ^24^ ^]^ To compare the SERS effects of Cu_2_O superparticle‐CP3 and Cu_2_O cube‐CP3, we immerse the two sample catalysts in 1 m K_2_CO_3_ solution and collect the corresponding Raman spectra (Figure S27, Supporting Information). It turns out that the peak intensities for adsorbed CO_3_^2−^ are identical for the two cases, implying that Cu_2_O superparticle‐CP3 and Cu_2_O cube‐CP3 have nearly the same SERS effect. Thus, the influence of SERS effects onthe peak intensities in the in situ Raman spectra can be excluded. We thus examine the effect of local pH on CO_2_RR in the case of two catalysts. In our measurement, the electrolyte is CO_2_‐saturated 0.1 m KHCO_3_ with a bulk pH of 6.8, in which carbonate is mainly present in the form of HCO_3_^−^. As such, higher pH values can shift the equilibrium to CO_3_^2−^, resulting in more intense peaks at 1070 cm^−1^.^[^ ^25^ ^]^

Given the information, we infer that Cu_2_O superparticle‐CP3 induces higher local pH than Cu_2_O cube‐CP3 during reaction. Furthermore, we recognize that as the potential becomes negative, the proton consumption increases leading to higher local pH. Intuitively the CO_3_^2−^ peak should become stronger; however, its intensity truly decreases beyond −0.75 V (vs RHE). Such a phenomenon, which has also been observed by several previous works,^[^ ^20^, ^23^, ^26^ ^]^ should be caused by the surface charge of Cu catalyst. At more negative potentials, the Cu catalyst surface becomes more negatively charged, hindering the adsorption of CO_3_^2−^. As a matter of fact, high local pH has been demonstrated as an important factor for accelerating C–C coupling.^[^ ^2^ ^]^ Under a condition of high local pH, sufficient OH^−^ species are located on or near the Cu surface, which can lower the energy barriers for *CO generation and C–C coupling.^[^ ^27^ ^]^ Moreover, high local pH suppresses HER, further enhancing the CO_2_RR selectivity. As such, the higher local pH, induced by Cu_2_O superparticle‐CP3, is believed to be responsible for the enhanced C–C coupling ability compared to Cu_2_O cube‐CP3.

To find out the origin of high local pH, an underpotential deposition of lead method is used to measure the electrochemical active surface area (ECSA) of the Cu electrocatalysts (Figure 5c; Figure S28, Supporting Information). The obtained ECSA is 1.227 cm^2^ for Cu_2_O superparticle‐CP3, very close to that of Cu_2_O cube‐CP3 (1.231 cm^2^). Moreover, the total reaction current densities of Cu_2_O superparticle‐CP3 and Cu_2_O cube‐CP3 are comparable at each tested potential (Figure 3d). These results indicate that the higher local pH for Cu_2_O superparticle‐CP3 over Cu_2_O cube‐CP3 is not attributed to the faster depletion of protons or production of hydroxide anions from the HER and CO_2_RR. We thus look into the unique superstructure of Cu_2_O superparticle‐CP3. The above observation by SEM and TEM has shown that large Cu aggregates are closely surrounded by dense 13.2 nm Cu nanoparticles, forming numerous nanoscale/sub‐nanoscale spacings between Cu facets. In sharp contrast, Cu_2_O cube‐CP3 lacks these nanogaps. A previous study has revealed that a porous Cu catalyst that can lead to higher local pH due to its ability to confine OH^−^ species in the nanogaps will present more pronounced OH^−^ electrosorption (OH_ads_) features.^[^ ^27b^ ^]^ As demonstrated in Figure 5d, Cu_2_O superparticle‐CP3 exhibits stronger (111), (100), and defect OH_ads_ features than Cu_2_O cube‐CP3. This implies that the superparticle‐derived catalyst can confine the OH^−^ species to induce higher local pH during reaction, which is consistent with the observation from in situ Raman spectroscopy. We thus infer that the generated OH^−^ can be concentrated and confined in these nanoscale/sub‐nanoscale gaps during reaction, leading to high local pH. To further prove this assumption, we compare the in situ Raman spectra and OH^−^ electrosorption for the Cu_2_O superparticles that have been pre‐electroreduced at −0.5 mA cm^−2^ (Cu_2_O superparticle‐CP0.5) and −3 mA cm^−2^ (Cu_2_O superparticle‐CP3). As discussed above, compared to pre‐electroreduction at −3 mA cm^−2^, more Cu_2_O building blocks fuse to form large Cu aggregates while substantially less particles are detached during the pre‐electroreduction at −0.5 mA cm^−2^. As a result, Cu_2_O superparticle‐CP0.5 contains a smaller number of nanoscale/sub‐nanoscale spacings between Cu facets, leading to relatively lower local pH. As shown in Figure S29 (Supporting Information), Cu_2_O superparticle‐CP0.5 shows weaker CO_3_^2−^ peak and OH_ads_ features than Cu_2_O superparticle‐CP3, supporting our argument. In addition, the decrease of C_2_H_4_ FE during the long‐term electrolysis at −40 mA cm^−2^ can be ascribed to the gradual loss of nanoscale/sub‐nanoscale gaps as small satellite particles slowly aggregate to form larger particles.

Taken together, due to the unique assembly structure of Cu_2_O superparticle, the building blocks in the outer shell detach during the pre‐electroreduction, resulting in numerous nanoscale/sub‐nanoscale spacings between Cu facets. During CO_2_ electroreduction, OH^−^ species generated from Cu surfaces diffuse to these nanoscale/sub‐nanoscale gaps. Due to the space confinement effect, further diffusion of OH^−^ species out of these nanoscale/sub‐nanoscale gaps becomes difficult.^[^ ^28^ ^]^ As such, OH^−^ species are concentrated in these nanoscale/sub‐nanoscale gaps, leading to a high local pH (Figure 5e). Combined with the advantage of grain boundary effect, Cu_2_O superparticle‐CP3 can achieve high selectivity for C_2+_ products. In contrast, although Cu_2_O cube‐CP3 offers similar grain boundaries with low‐coordinated Cu atoms and ECSA, it cannot provide the capability of confining OH^−^ species to achieve high local pH due to the absence of nanoscale/sub‐nanoscale gaps, showing lower C–C coupling ability (Figure 5f).

## 3 Conclusion

In summary, we have proposed a concept that Cu_2_O superparticles can serve as a precatalyst for the formation of a unique “planet–satellite”‐like Cu structure via pre‐electroreduction by a CP method. Our electron microscopic characterization together with in situ XAS and Raman spectroscopy have revealed that during the structural reconstruction, the building blocks inside the superparticle fuse to generate numerous Cu grain boundaries while those in the outer shell detach to form nanogap structures that can efficiently confine OH^−^ for sustaining high local pH. Such a unique superstructure combines the advantages of grain boundaries and high local pH to C−C coupling, which can otherwise be impossible to attain from other Cu‐based precatalysts such as the widely explored Cu_2_O cube. Ultimately, the Cu_2_O superparticle‐derived Cu catalyst has achieved high FEs of 53.2% for ethylene and 74.2% for C_2+_ products, surpassing the performance of most reported Cu electrocatalysts. This work provides a new avenue based on superparticles for the development of advanced electrocatalysts, and also emphasizes the importance of rationally controlling precatalyst structure to the design of catalytically active sites.

## 4 Experimental Section

### Materials

Polyvinyl PVP (K30 and MW = 8000) and copper(II) acetylacetonate were purchased from Aladdin. Oleylamine (70%) was purchased from Sigma–Aldrich. Ketjen black carbon (ECP600JD) was purchased from LION Co., Ltd (Japan). Commercial Cu particles (150 nm, sphere shape) were purchased from Shanghai Xiangtian nanomaterials Co., Ltd. Other reagents were purchased from Sinopharm Chemical Reagent Co., Ltd. Deionized water (18.25 MΩ cm) was used in all experiments. All the chemical reagents were used as received without any further purification.

### Preparation of Cu_2_O Superparticles

Cu_2_O superparticles were prepared using a modified method from a previous report.^[^ ^11^ ^]^ In a typical synthesis, 1.5 mmol of Cu(NO_3_)_2_·3H_2_O, 3.0 g of PVP (MW = 8000), and 30 mL of diethylene glycol (DEG) were added into a three‐necked flask (50 mL). The flask was then sealed and degassed by vacuum for ≈15 min. After Cu (NO_3_)_2_·3H_2_O and PVP were dissolved in DEG completely, argon was introduced into the flask to form an inert environment. The mixed solution was heated from room temperature to 190 °C in ≈30 min and cooled naturally. The products were collected by centrifugation and washed with water and ethanol three times, respectively. After further drying in a vacuum chamber at 60 °C, the product was stored in a vacuum storage tank.

### Preparation of Cu_2_O Monocrystalline Cubes

Cu_2_O cubes were synthesized according to a previous method.^[^ ^29^ ^]^ Deionized water (DI water) was used throughout the experiment and the room temperature was ≈22 °C. Fifty milligrams of cupric acetate and 270 mg of PVP‐K30 were added to 100 mL of DI water. The solution was stirred at 700 rpm until forming a clear blue solution. Then 20 mL of NaOH solution (0.25 m) was added dropwise into the blue solution. After reaction for ≈7 min, 15 mL of ascorbic acid (0.05 m) was added drop by drop and the resultant solution was kept under stirring for 30 min. The products were centrifuged immediately, and then washed with DI water and ethanol for two times, respectively. After further drying in a vacuum chamber at 60 °C, the product was stored in a vacuum storage tank.

### Working Electrode Preparation

The working electrode was prepared based on the precatalyst ink. Five milligrams of Cu_2_O superparticles, 2.5 mg of Ketjen black carbon, and 30 µL of Nafion solution (5 wt.%) were dispersed in 970 µL of isopropanol by sonicating for at least 30 min to form a homogeneous ink. Then 10 µL of the ink was uniformly spread on a glass carbon electrode (5 mm in diameter) and dried. To prepare working electrodes for the Cu_2_O cube and commercial Cu particles, the procedure was the same.

### Electrocatalytic Measurement

Electrocatalytic CO_2_ reduction was carried out in a typical gas‐tight H‐type cell. The two counterparts were separated by Nafion 117 membrane. A Pt foil of 1 cm^2^ and the saturated Ag/AgCl electrode were used as the counter electrode and reference electrode, respectively. The electrochemical measurements were controlled by the electrochemical workstation (CHI660E). All the working potentials were referred to RHE based on the following equation:

(1) $$E\;\left(\mathrm{vs}\;\mathrm{RHE}\right)=E\;\left(\mathrm{vs}\;\mathrm{Ag}/\mathrm{AgCl}\right)+0.197\;V+0.0592\times \mathrm{pH}$$

Before electrocatalytic CO_2_ reduction measurement, both cathode and anode sides were filled with 40 mL of 0.1 m KHCO_3_ aqueous solution. The headspace volume of the cathode side was 32 mL. Then high‐purity CO_2_ (99.999%) was bubbled into the electrolyte for at least 20 min to obtain CO_2_‐saturated 0.1 m KHCO_3_ (pH 6.8), after which the cell was sealed. After pre‐electroreduction at −3 mA cm^−2^ for 1000 s, the electrolyte in the cathode side was recovered and saturated by high purity CO_2_ again. Controlled potential electrolysis was performed by the electrochemical workstation with a factor of 85% for iR‐compensation. The electrolysis at each fixed potential was conducted at least 40 min to accumulate enough products for accurate detection. Specifically, the electrolysis time was 2.5 h for −0.85 V, 1.5 h for −0.95 V, 1 h for −1.05 V and −1.15 V, and 40 min for −1.25 V (vs RHE). The electrolysis at each potential was repeated at least three times on different fresh electrodes. For stability test, the electrolysis was conducted at a constant current density of −40 mA cm^−2^. High‐purity CO_2_ was continuously poured into the electrolyte in the cathodic compartment at 10 sccm. The gas products were collected using a gas bag, and the electrolyte was recovered every 6 h.

### Product Analysis

After each electrolysis, the gaseous products in the headspace of the cathode side or the gas bag were analyzed by the gas chromatography (GC, 7890A and 7890B, Ar carrier, Agilent). The sampled gas was separated by three packed columns. H_2_ was detected by thermal conductivity detector (TCD). CO was converted to CH_4_ by a methanizer and then analyzed by flame ionization detector (FID). CH_4_ and C_2_H_4_ were analyzed by another FID. The gas product quantification was determined using a serious of standard curves of H_2_, CO, CH_4,_ and C_2_H_4_. For liquid products analysis, ^1^H NMR (Bruker AVANCE AVIII 400) was used. Five hundred milliliters of catholyte after electrolysis was mixed with 100 µL of D_2_O and 0.02 µL of dimethyl sulfoxide (DMSO). DMSO was added as an internal standard. The 1D ^1^H spectrum was measured with water suppression. The faradaic efficiency of each product was calculated using the following equation:

(2) $$\mathrm{FE}\;\left(\%\right)=\left(n_{e}\times n\times F\right)/Q\times 100\%$$

where *F* is the Faraday constant (96 485 C mol^−1^); *n* is the mole amount of the product; *n*_e_ is the number of electrons that are needed to produce one molecule of product (*n*_e_ = 2, 2, 2, 6, 8, 12, 12, and 18 for H_2_, CO, HCOOH, CH_3_OH, CH_4_, C_2_H_4_, C_2_H_5_OH, and *n*‐C_3_H_7_OH, respectively); *Q* is the total amount of charge (in units of coulombs) passed through the working electrode.

## Conflict of Interest

The authors declare no conflict of interest.

## Supporting information

Supporting Information

## Data Availability

The data that support the findings of this study are available in the supplementary material of this article.
