# Supplementary material for: Structural Reconstruction of Cu2O Superparticles toward Electrocatalytic CO2 Reduction with High C2+ Products Selectivity
Source: Adv Sci (Weinh). 2022 Apr 1;9(16):2105292. doi: 10.1002/advs.202105292 (PMC9165515; doi:10.1002/advs.202105292)
Supplement: Supplementary file 1 — Supporting Information [file ADVS-9-2105292-s001.pdf]

© 2022 Wiley-VCH GmbH

Supporting Information

Structural Reconstruction of Cu_2O Superparticles toward Electrocatalytic CO_2 Reduction with High C_{2+} Products Selectivity

Yawen Jiang, Xinyu Wang, Delong Duan, Chaohua He, Jun Ma, Wenqing Zhang, Hengjie Liu, Ran Long, Zibiao Li, Tingting Kong, Xian Jun Loh, Li Song, Enyi Ye,* and Yujie Xiong**

Y. Jiang,[+] X. Wang,[+] D. Duan,[+] C. He, J. Ma, W. Zhang, Dr. H. Liu, Prof. R. Long, Prof. L. Song, Prof. Y. Xiong
Hefei National Laboratory for Physical Sciences at the Microscale
Frontiers Science Center for Planetary Exploration and Emerging Technologies
School of Chemistry and Materials Science
National Synchrotron Radiation Laboratory
University of Science and Technology of China
Hefei, Anhui 230026, China
E-mail: longran@ustc.edu.cn; yjxiong@ustc.edu.cn

Dr. Z. Li, Dr. X. J. Loh, Dr. E. Ye
Institute of Materials Research and Engineering
A*STAR (Agency for Science, Technology and Research)
2 Fusionopolis Way, Innovis, #08-03, 138634, Singapore
Email: yeey@imre.a-star.edu.sg

Prof. T. Kong
College of Chemistry and Chemical Engineering
Xi'an Shiyou University
Xi'an, Shaanxi 710054, China

[+] These authors contributed equally.

Experimental Section

OH^- adsorption reaction: OH^- adsorption reaction cyclic voltammetry (CV) was conducted in 0.1 M KOH solution at a 100 mV/s scan rate after the pre-electroduction of the Cu_2O catalysts. Ar was poured into 0.1 M KOH solution for at least 20 min to eliminate the dissolved oxygen before test.

Lead Under Potential Deposition (Pb-UPD): Electrochemical active surface area (ECSA) was measured by an underpotential deposition of lead on Cu surface. After pre-electroreduction at −3 mA/cm^2, the electrodes were transferred into an Ar-saturated aqueous solution of 0.01 M $Pb(ClO_4)_2$ and 0.1 M $HClO_4$. Then CV was measured within 0 V to −0.4 V vs the saturated Ag/AgCl reference electrode in a scan rate of 10 mV/s. The area of a Pb monolayer stripping peak of the final CV was integrated to calculate the transferred charge. The conversion factor of 300 μC cm^{-2} was used assuming a hexagonal closed packed Pb monolayer formed on Cu surfaces in a 2-electron process.[1]

In situ X-ray absorption spectroscopy (XAS): The in situ XAS measurement under the sensitive fluorescence model was carried out at Beamline 1W1B at BSRF. The catalyst ink was dispersed on a piece of gas diffusion electrode (GDL, YLS-30T). Several pieces of copper tape and Kapton tape were used to fix the working electrode on the exterior wall of the in situ electrochemical cell. A carbon rod and a saturated Ag/AgCl electrode were used as the counter electrode and reference electrode, respectively. CO_2-saturated 0.1 M $KHCO_3$ was circulated at 1 mL/min through the cell with the assistance of a variable-speed peristaltic pump. Before the measurement, the solution resistance (R) was tested, and a factor of 85% for iR compensation was done manually after the test. The potential during the in situ characterization was controlled to be about −1.15 V (vs RHE). The XAS data was processed using the Athena and Artemis programs.

In situ Raman measurement: In situ Raman spectra were taken on a WITec alpha 300R confocal Raman microscope with a 600 grooves/mm diffraction grating. A 633 nm excitation laser was used with an exact power of 5 mW. Calibration was performed based on the peak at 520 cm^{-1} of a silicon wafer standard. The catalyst ink (0.5 mg Ketjen black carbon added) was dispersed on a glass carbon electrode (5 mm in diameter). A Pt wire and a saturated Ag/AgCl electrode were used as the counter electrode and reference electrode, respectively. Before the measurement, 0.1 M $KHCO_3$ was saturated by high-purity CO_2 and then added into the cell. High-purity CO_2 was continuingly introduced into the electrolyte and removed

once the signal was acquired. The Raman spectra were obtained at about 10 min after the potential was initially applied.

Sample characterizations: Scanning electron microscopy (SEM) images were taken on the Gemini SEM 500 and Carl Zeiss Supra 40 scanning electron microscope. Transmission electron microscopy (TEM) images were collected using the Talos F200X, Hitachi H-7650 and Hitachi HT7700 transmission electron microscope. Scanning transmission electron microscopy (STEM) images, high-resolution TEM (HRTEM) images and energy-dispersive X-ray spectroscopy (EDS) mapping profiles were collected on a JEOL JEM-2100F field-emission high-resolution transmission electron microscope. Selected area electron diffraction (SAED) patterns were recorded on a JEM-2010 transmission electron microscope. The Cu_2O superparticle was cut by a FEI Helios Nanolab650 focused ion beam (FIB). Powder X-ray diffraction (XRD) patterns were recorded using a a Philips X'Pert Pro Super X-ray diffractometer with Cu-Kα radiation (λ = 1.5418 Å). Fourier transform infrared (FTIR) spectroscopy was performed using a Thermo Nicolet 6700 spectrometer.

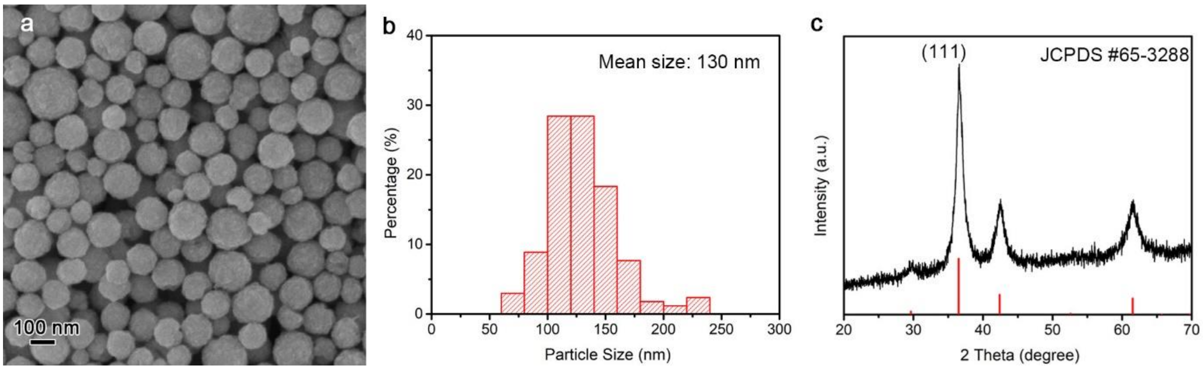

Figure S1. a) SEM image of the prepared Cu_2O superparticle. b) Size distribution of the Cu_2O superparticle. c) XRD pattern of the Cu_2O superparticle.

The peaks of XRD pattern in Figure S1c are consistent with those for face-centered cubic (fcc) Cu_2O (JCPDS #65-3288). The calculation for the strongest peak (111) using the Debye–Scherrer formula gives the grain size of 7.7 nm. This indicates that the Cu_2O building blocks are nanosized.

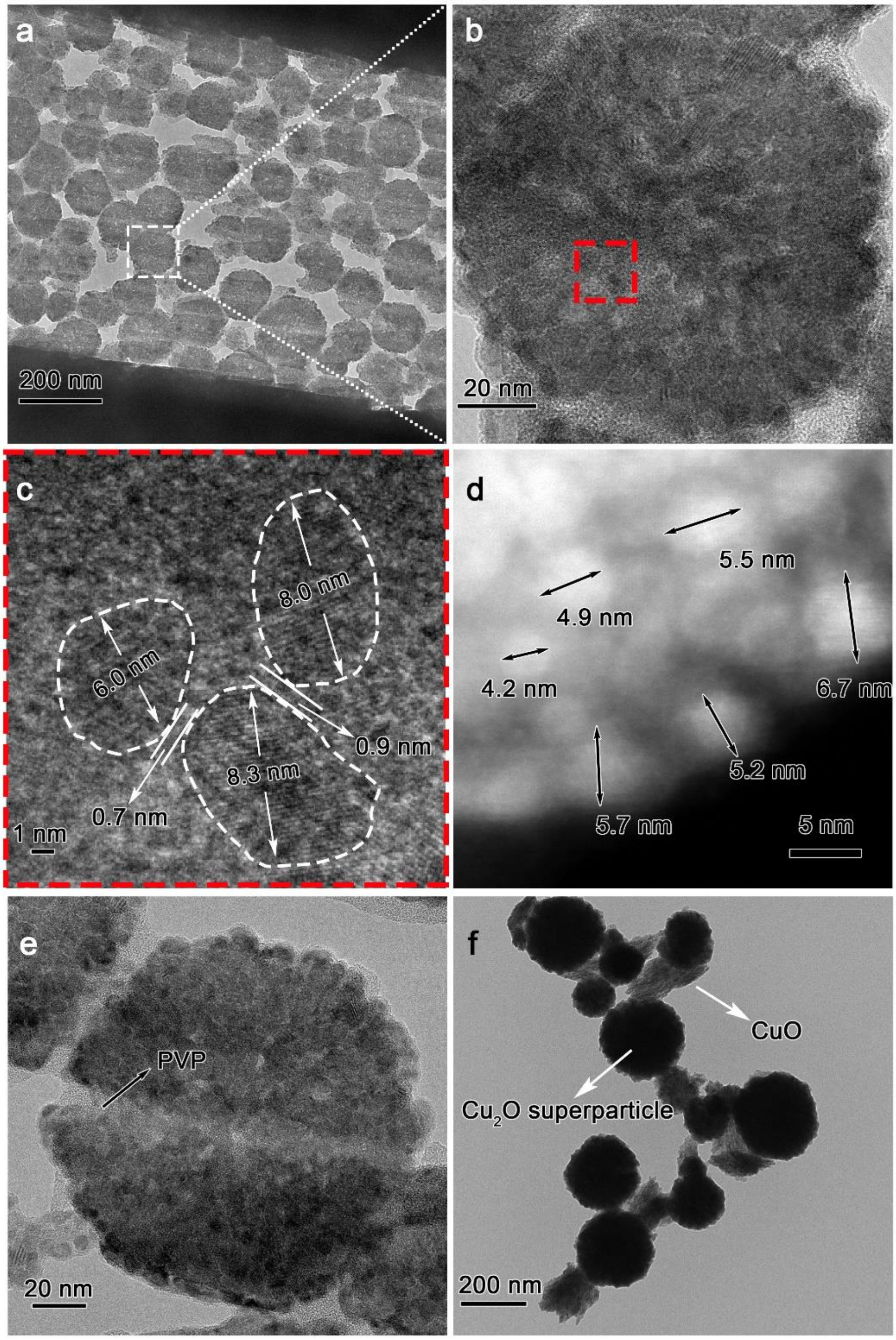

Figure S2. a, b) TEM images of the Cu_2O superparticle cut by FIB. c) Enlarged TEM image corresponding to the red square region of b). d) STEM image of Cu_2O superparticle. e) TEM image of the Cu_2O superparticle whose assemably structure is broken by FIB. f) TEM image of the Cu_2O superparticle after being exfoliated by ultrasonic cell pulverizer for 12 h.

The Cu_2O superparticle has a relatively large size of 130 nm. FIB technique is used to cut the Cu_2O superparticle into pieces with thickness of tens of nanometers so that TEM can observe the internal structure. From Figure S2c and S2d, we can clearly see that the Cu_2O superparticle is composed of many smaller particles (4-9 nm in size). The interspace between two building blocks is sub-1 nm so that van der Waals forces exist between the building

blocks. Figure S2e shows the image of a Cu_2O superparticle whose assembly structure is broken by FIB, indicating that PVP also exists inside the superparticle. This reveals that the building blocks are also covered by PVP which can act as bridging agents to induce the integration of neighboring nanoparticles.

20 mg of Cu_2O superparticles are dispersed in isopropanol, and then are exfoliated by ultrasonic cell pulverizer for 12 h. According to the TEM image (Figure S2c), the Cu_2O superparticle maintains its assembly structure without the building blocks separated. In addition, we note that after such a long time of exfoliation, the yellow solution turns a little dark, suggesting that a part of the Cu_2O superparticle has been oxidized to CuO.

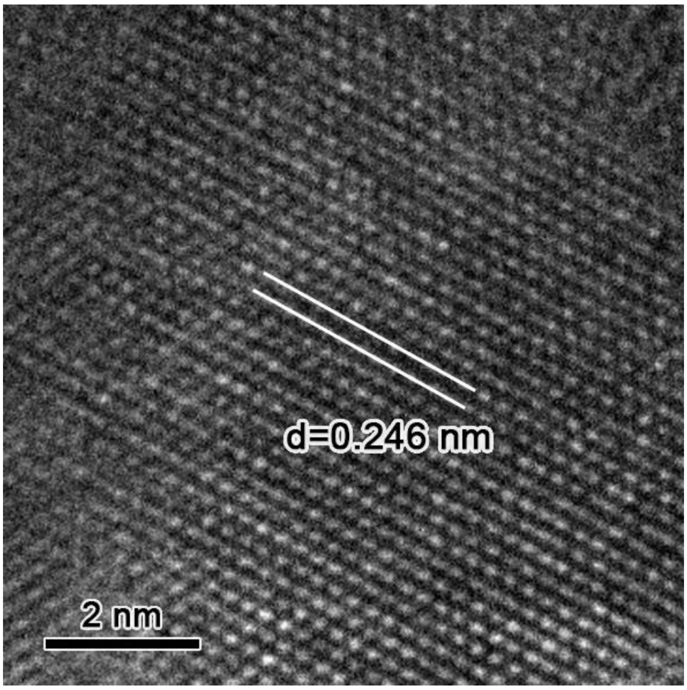

Figure S3. HRTEM of the prepared Cu_2O superparticle.

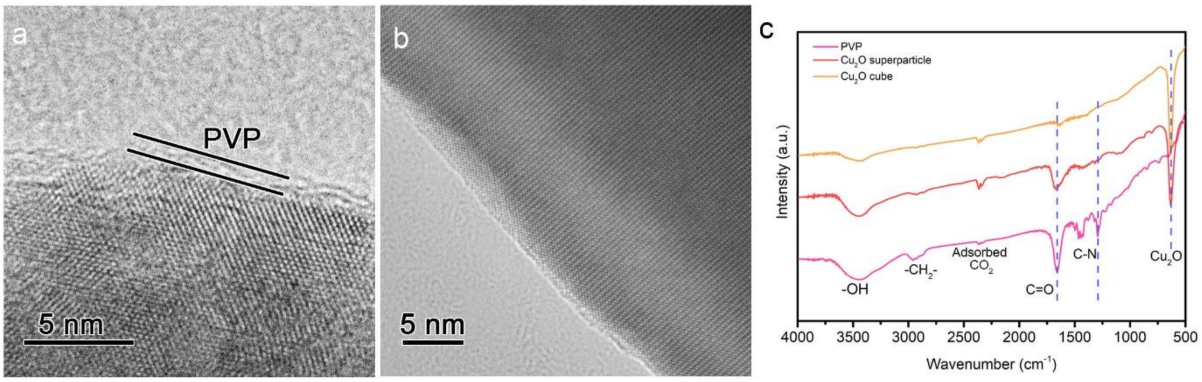

Figure S4. a) HRTEM image of the Cu_2O superparticle, suggesting that the surface is covered with a thin layer of PVP. b) HRTEM image of the Cu_2O cube, showing a cleaner surface. c) FTIR spectra of PVP, the Cu_2O superparticle and the Cu_2O cube.

A thin layer of PVP is observed on the surface of the Cu_2O superparticle (Figure S4a). Further analysis with FTIR (Figure S4c) also confirms the presence of surface-adsorbed PVP.

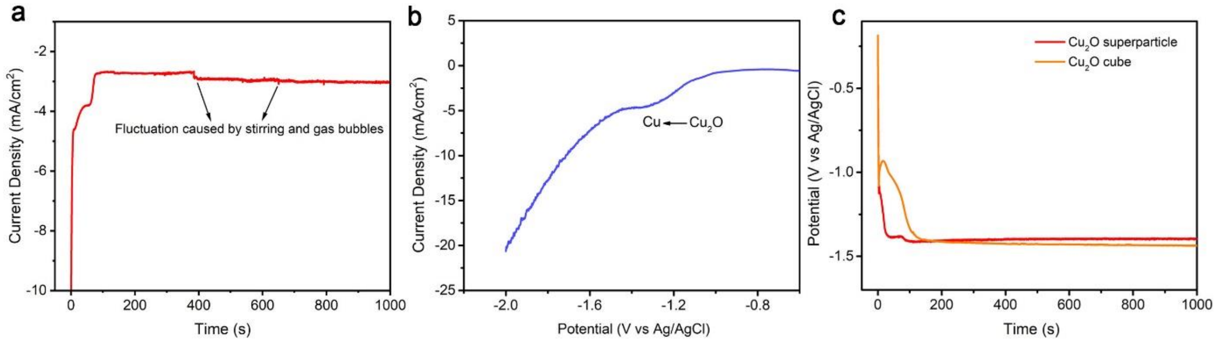

Figure S5. a) Current density vs time recorded during reduction at –1.37 V (vs Ag/AgCl) of Cu_2O superparticle in CO_2-saturated 0.1 M $KHCO_3$ solution. The steady current density is about –3.0 mA/cm^2. b) Linear sweep voltammetry (LSV) curve of the Cu_2O superparticle in CO_2-saturated 0.1 M $KHCO_3$ solution without iR compensation. c) Potential vs time recorded during pre-electroreduction at −3.0 mA/cm^2 of the Cu_2O superparticle and the Cu_2O cube in CO_2-saturated 0.1 M $KHCO_3$ solution.

Compared to pre-electroreduction at a fixed current, the response current at a fixed potential is more susceptible to stirring and gas bubbles (Figure S5a), making it difficult to judge the steady state sometimes. For this reason, chronopotentiometry is used for the pre-electroreduction of the Cu_2O catalysts.

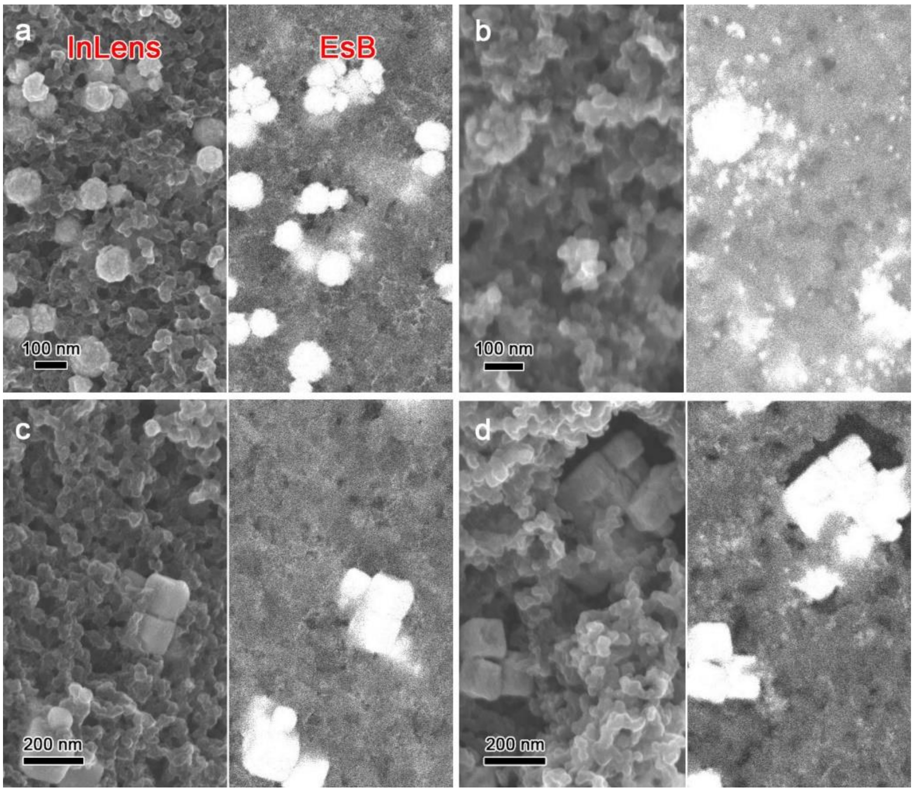

Figure S6. SEM images of the Cu_2O superparticle a) before and b) after the pre-electroreduction. SEM images of the Cu_2O cube c) before and d) after the pre-electroreduction. For each image, the left is the InLens signal while the right is the EsB signal of the same zone.

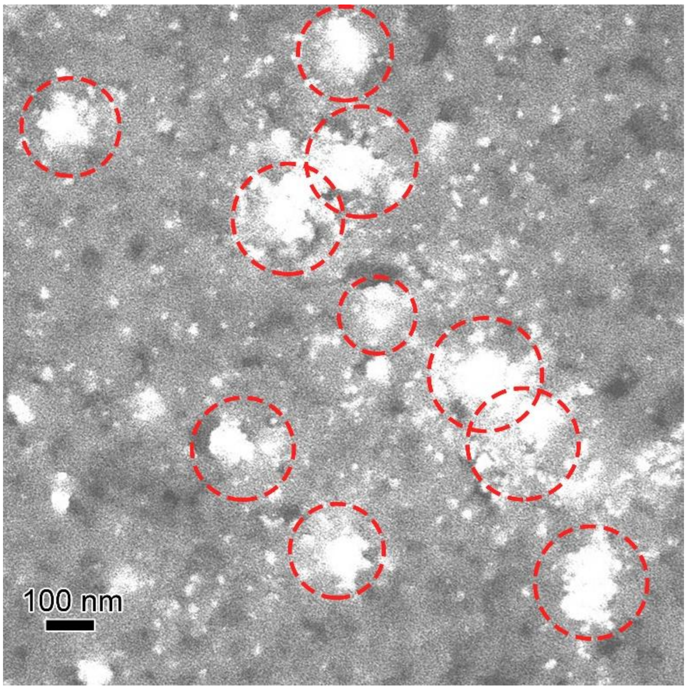

Figure S7. SEM image obtained by the EsB detector of Cu_2O superparticle-CP3. The red circles indicate the "planet-satellite" like structure, in which the large aggregates are closely surrounded by dense small nanoparticles.

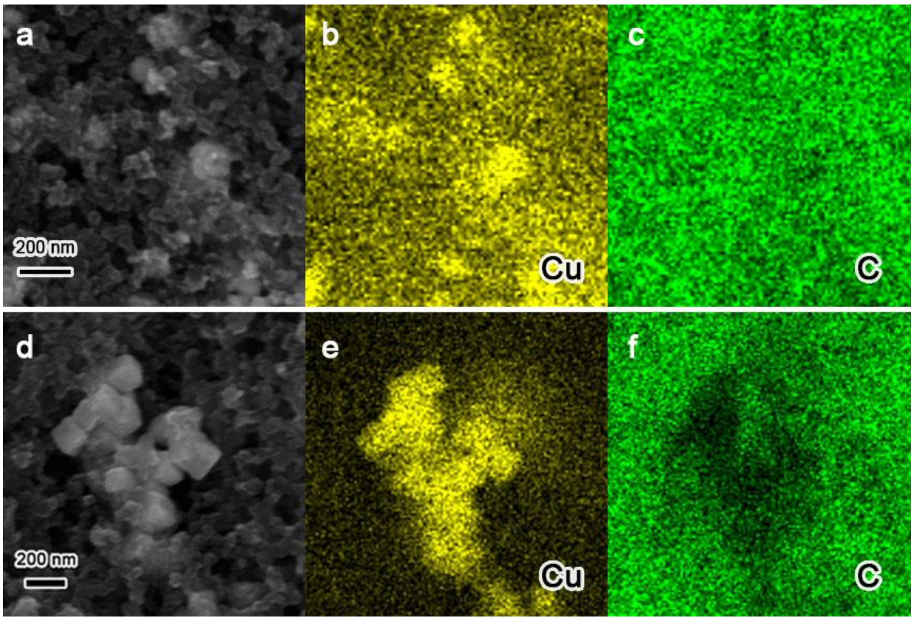

Figure S8. SEM-EDS elemental mapping for a–c) Cu_2O superparticle-CP3 and d–f) Cu_2O cube-CP3.

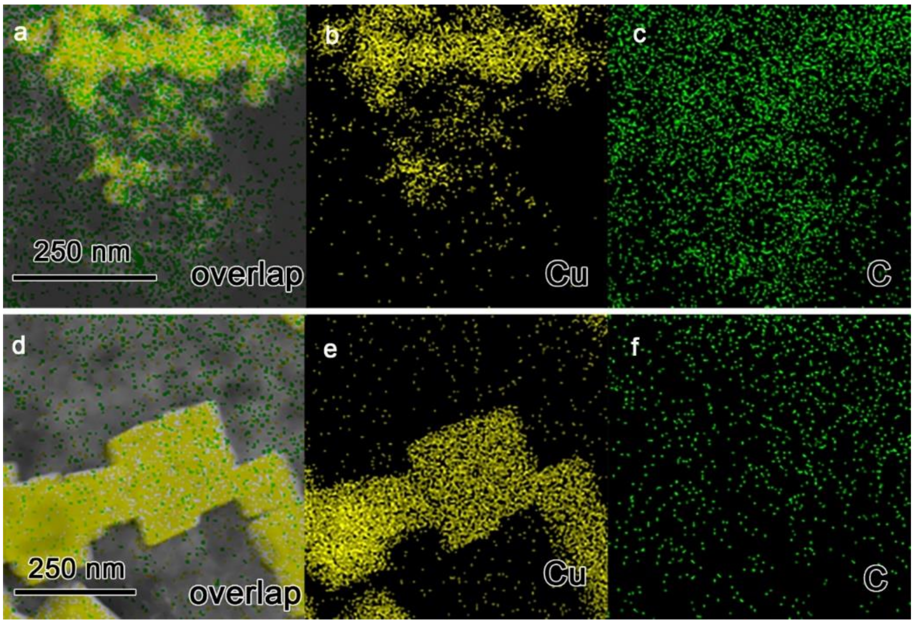

Figure S9. STEM-EDS elemental mapping for a–c) Cu_2O superparticle-CP3 and d–f) Cu_2O cube-CP3.

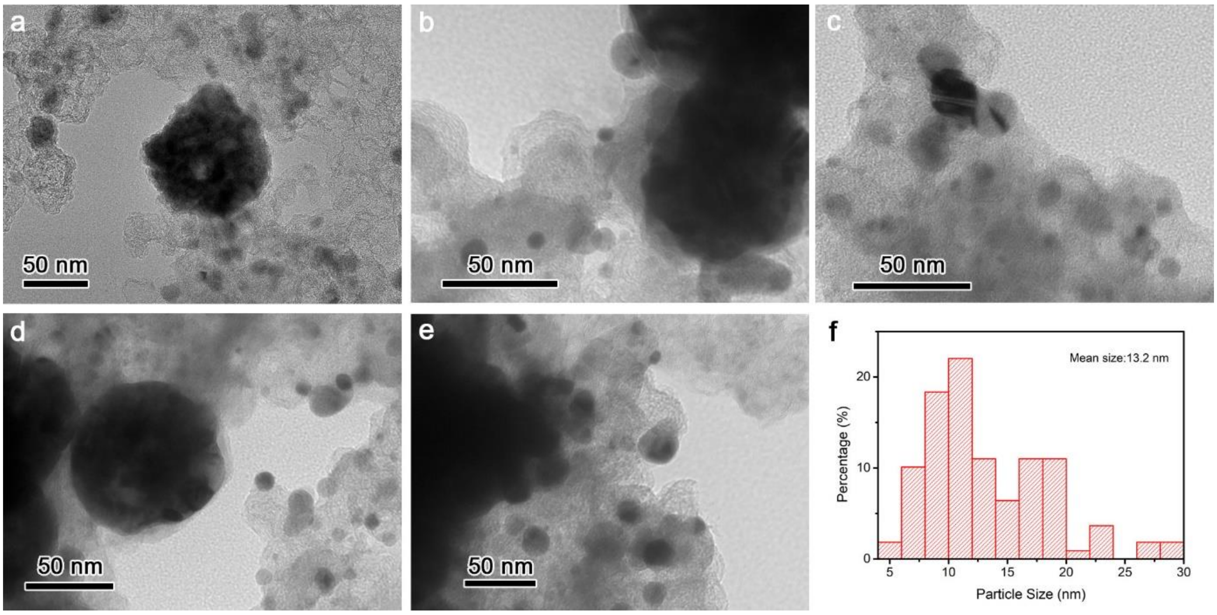

Figure S10. a–e) TEM images of small rounded particles around the large aggregates. f) Size distribution of the small rounded nanoparticles.

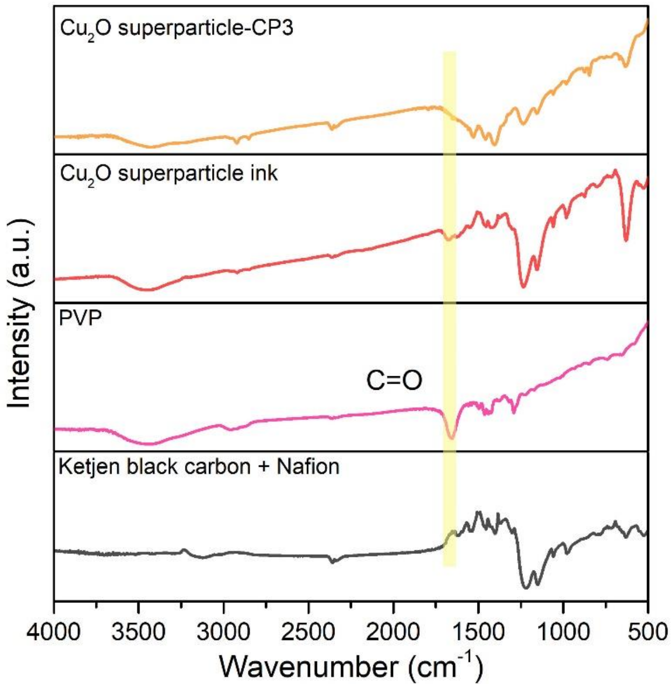

Figure S11. FTIR spectra of Ketjen black carbon + Nafion, PVP, dried Cu_2O superparticle ink and Cu_2O superparticle-CP3.

It is noted that too much Ketjen black carbon in the catalyst ink will block all the IR signal. In order to avoid this problem, we reduce the amount of Ketjen black carbon to 0.5 mg in the ink. After several times of pre-electroreduction, the catalysts peeled off from the glass carbon electrode (10 mm in diameter) are characterized by FTIR. As shown in the FTIR spectra, the peak belonging to C=O of PVP can be seen in the spectrum of the dried Cu_2O superparticle ink. After the pre-electroreduction, this peak disappears, suggesting that PVP has been stripped off during the electroreduction process.

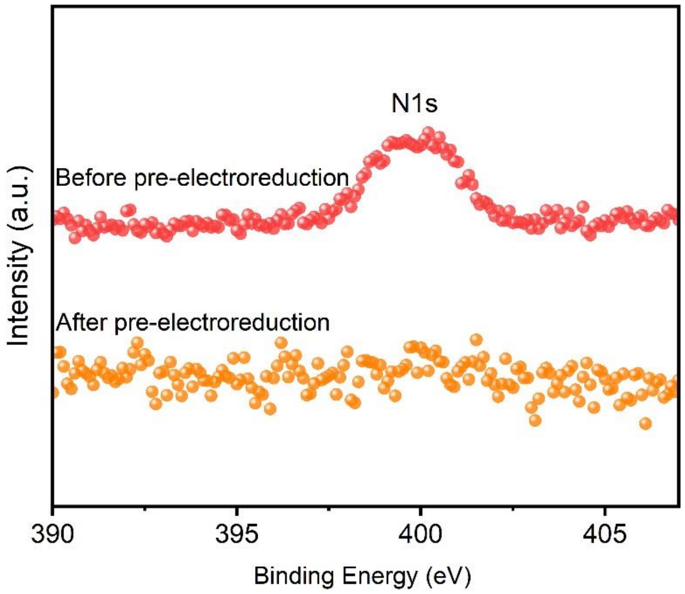

Figure S12. N1s XPS spectra of Cu_2O superparticle before and after pre-electroreduction at –3 mA/cm^2.

The N1s signal originates from the surface adsorbed PVP. After pre-electroreduction, we cannot detect the N1s signal, suggesting that PVP has been stripped off.

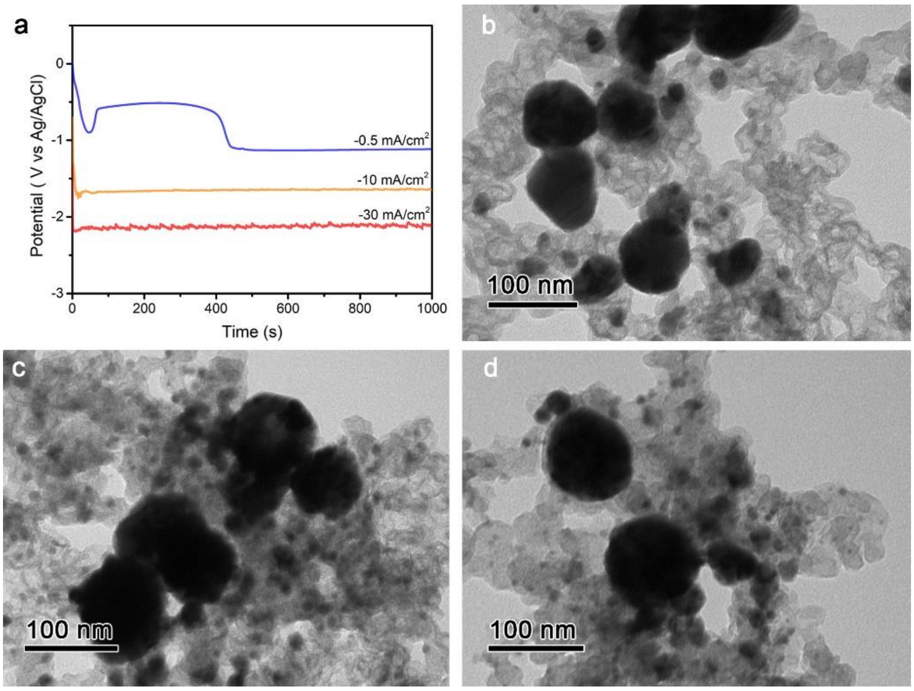

Figure S13. a) Potential vs time recorded during pre-electroreduction of the Cu_2O superparticle at different current densities. TEM images of Cu_2O superparticle after pre-electroreduction at different current densities: b) −0.5 mA/cm^2, c) −10 mA/cm^2, d) −30 mA/cm^2.

When the Cu_2O superparticle is reduced at a very low current density of −0.5 mA/cm^2 (further reducing current density does not reduce Cu_2O according to Figure S5a), the building blocks are more favorable to fuse to form large aggregates while substantially less small particles are detached. Such a phenomenon is caused by the different reduction rate of Cu_2O. As metallic Cu has a smaller unit cell than Cu_2O, the volume of the Cu_2O building blocks will become smaller during the reduction. If the reduction goes very slowly, the volume of the building blocks will also change slowly. As a result, small particles in the outer region are more likely to aggregate with the inner particles. In contrast, if reduction rate is relatively fast, the volume of the building blocks in the outer region will turn smaller quickly so that they are detached from the inner ones.

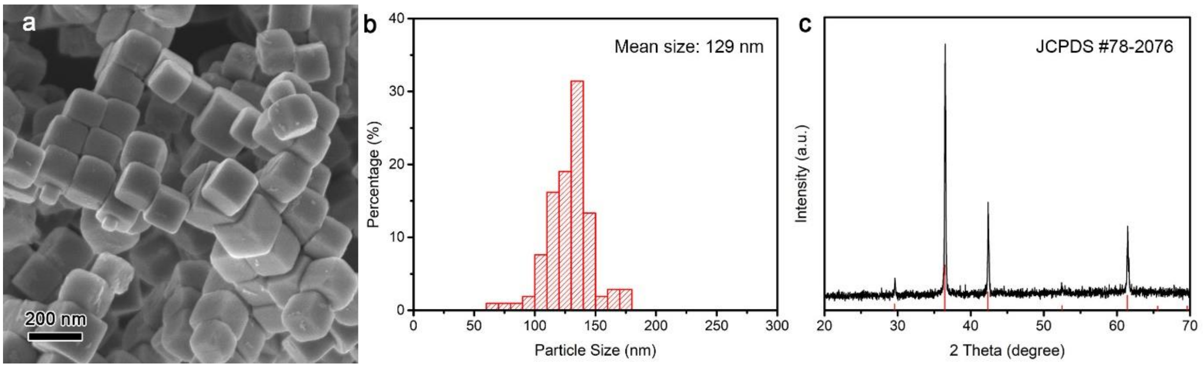

Figure S14. a) SEM image of the prepared Cu_2O cube. b) Size distribution of the Cu_2O cube. c) XRD pattern of the Cu_2O cube.

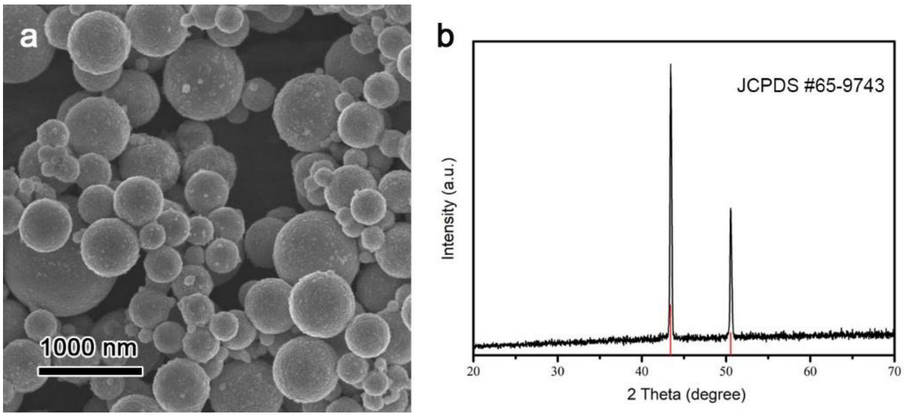

Figure S15. a) SEM image of commercial Cu particles. b) XRD pattern of commercial Cu particles.

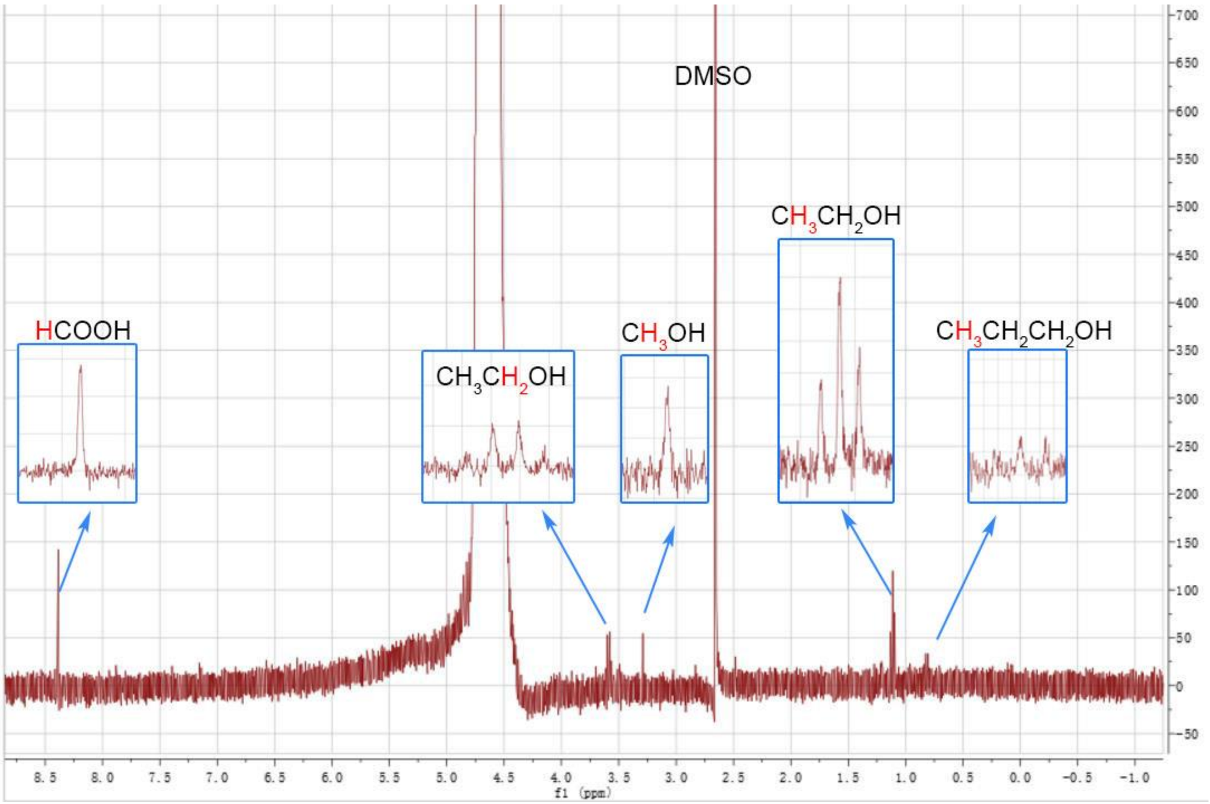

Figure S16. A typical ^{1}H NMR spectrum of the detected liquid products.

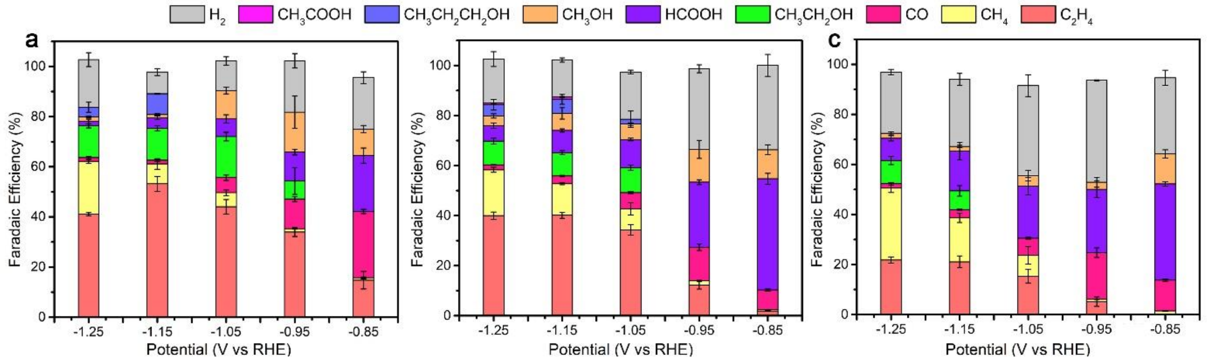

Figure S17. Potential-dependent FEs of all the detected products on a) Cu_2O superparticle-CP3, b) Cu_2O cube-CP3 and c) commercial Cu particles.

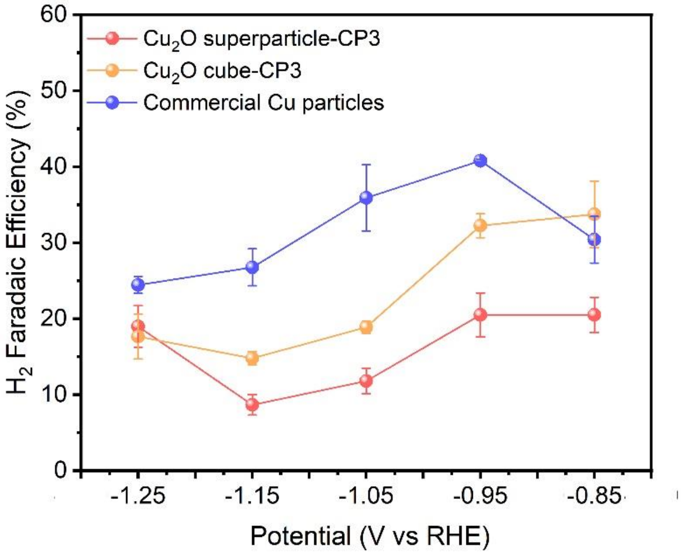

Figure S18. Potential-dependent FEs of H_2 on Cu_2O superparticle-CP3, Cu_2O cube-CP3 and commercial Cu particles.

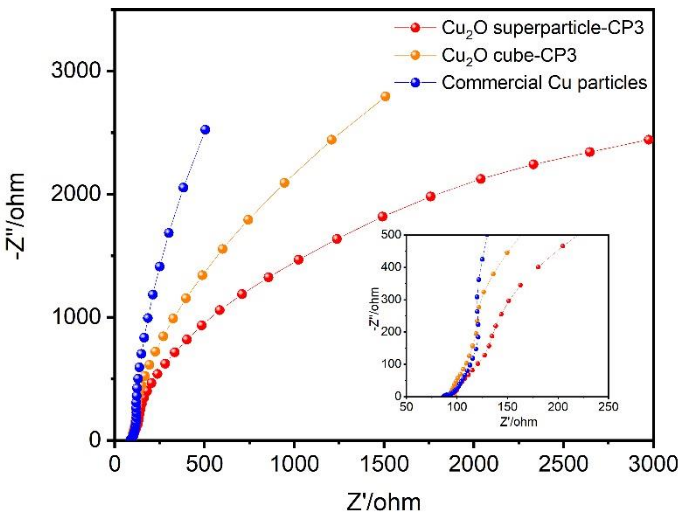

Figure S19. Nyquist plots of the three types of catalysts in CO_2-saturated 0.1 M $KHCO_3$ electrolyte at the open circuit potentials.

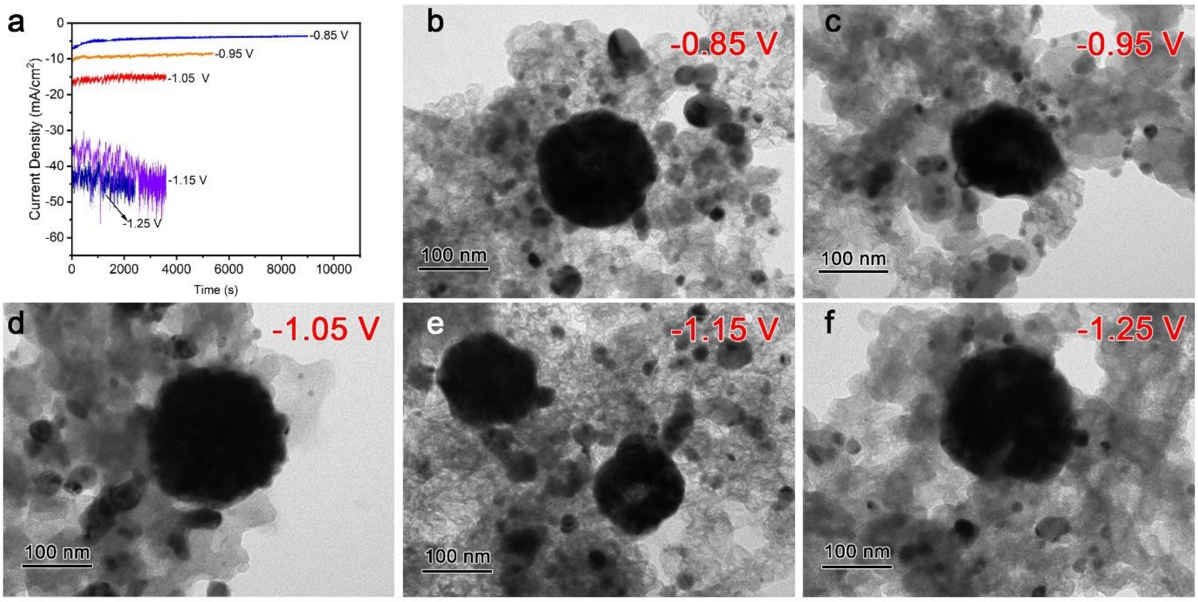

Figure S20. a) Measured chronoamperometry curves during reactions. TEM images of Cu_2O superparticle-CP3 after reaction at different potentials: b) –0.85 V (vs RHE), c) –0.95 V (vs RHE), d) –1.05 V (vs RHE), e) –1.15 V (vs RHE) and f) –1.25 V (vs RHE).

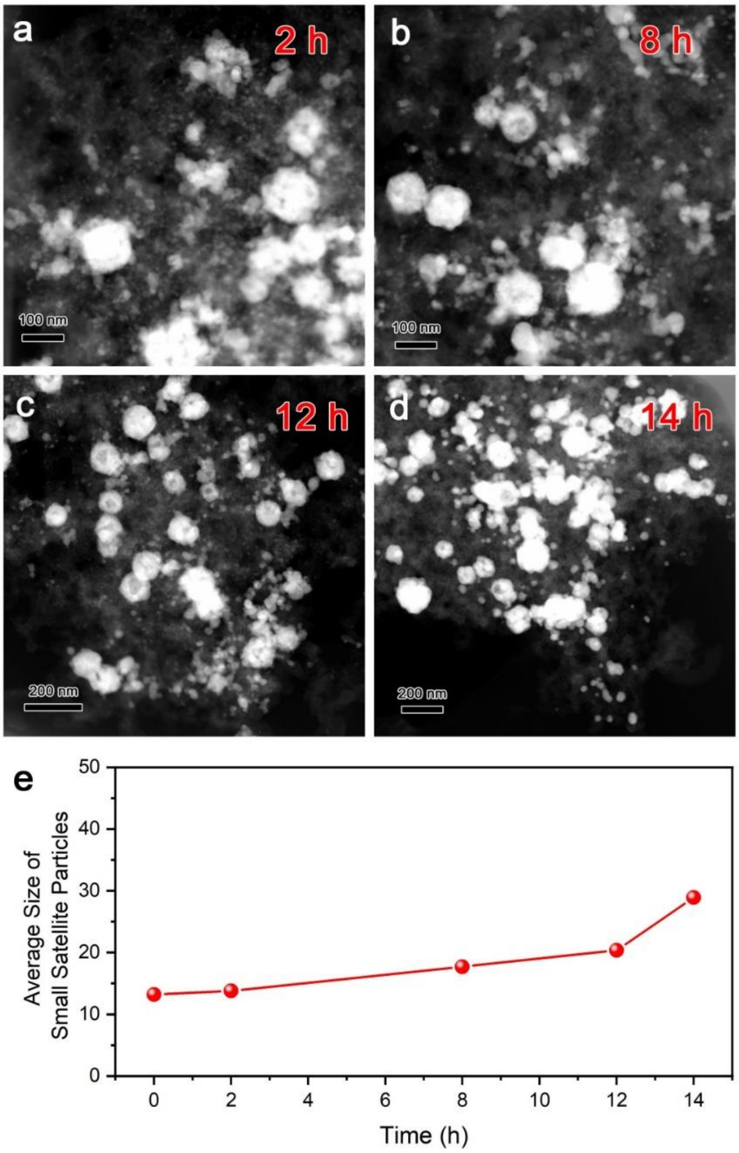

Figure S21. STEM images of Cu_2O superparticle after reaction at –40 mA cm^{-2} for different time: a) 2 h, b) 8 h, c) 12 h and d) 14 h. e) The average size of small satellite particles in Cu_2O superparticle-CP3 after reaction at –40 mA cm^{-2} for different time.

The growth of the small satellite particle size is the result of sintering during long-time electrolysis. It has been reported that applied potential and reaction intermediates can induce the structural reconstruction of electrocatalysts, especially for nanoparticles with small size.[2] The small satellite particles with high surface energies are relatively unstable during electrocatalytic reaction. They will undergo diffusion, collision and coalescence, resulting in an increase in size, which is consistent with previous reports.[3]

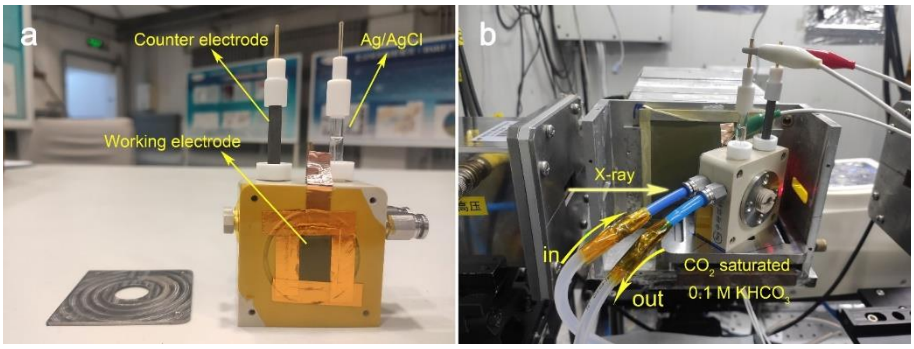

Figure S22. a) The electrochemical cell for in situ XAS measurement. b) The experimental setup for in situ XAS measurement.

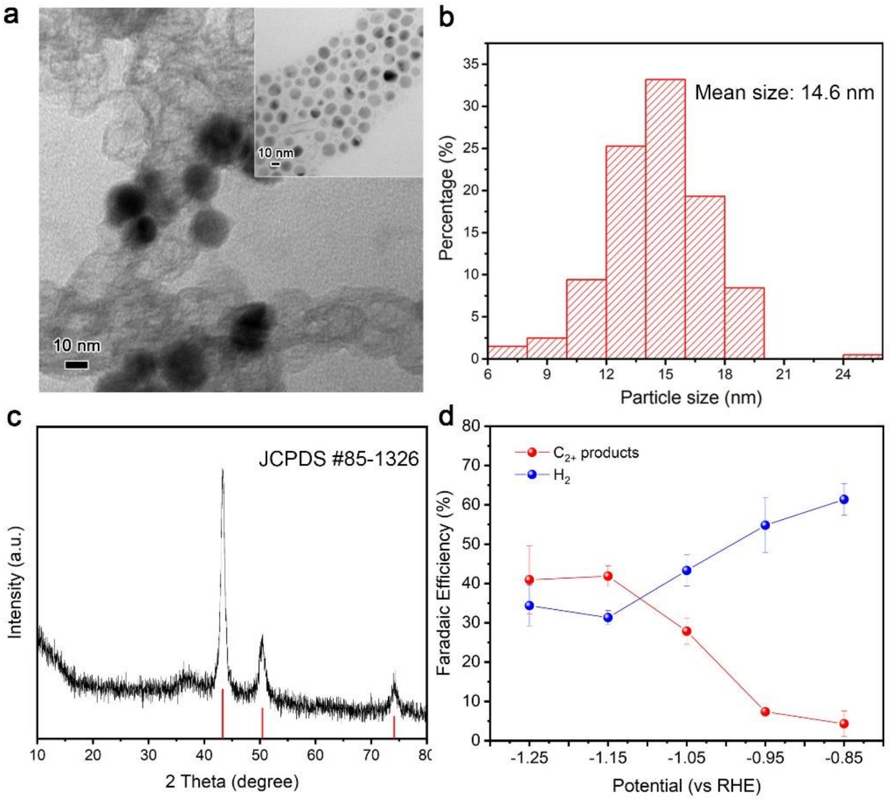

Figure S23. a) TEM images, b) size distribution and c) XRD pattern of the prepared 14.6 nm Cu rounded nanoparticles. d) Potential-dependent FEs of C_{2+} products and H_2 for 14.6 nm Cu nanoparticles.

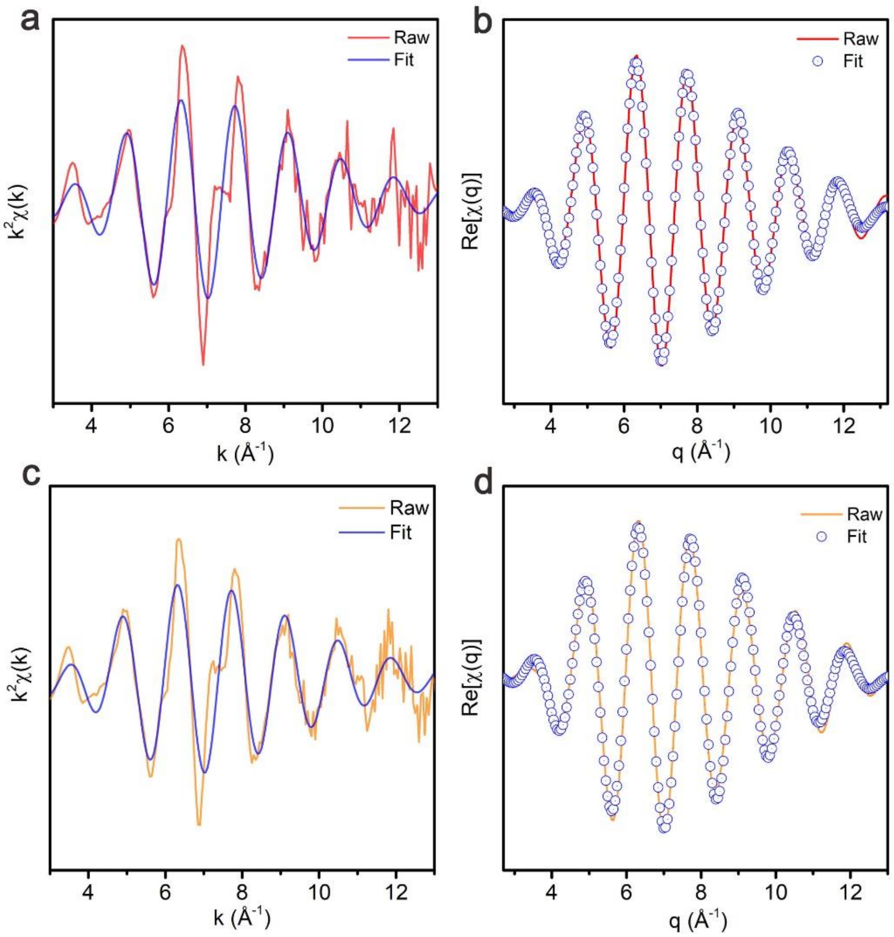

Figure S24. EXAFS fitting curves in a) k space and b) q space of Cu_2O superparticle-CP3 at −1.15 V (vs RHE). EXAFS fitting curves in c) k space and d) q space of Cu_2O cube-CP3 at −1.15 V (vs RHE).

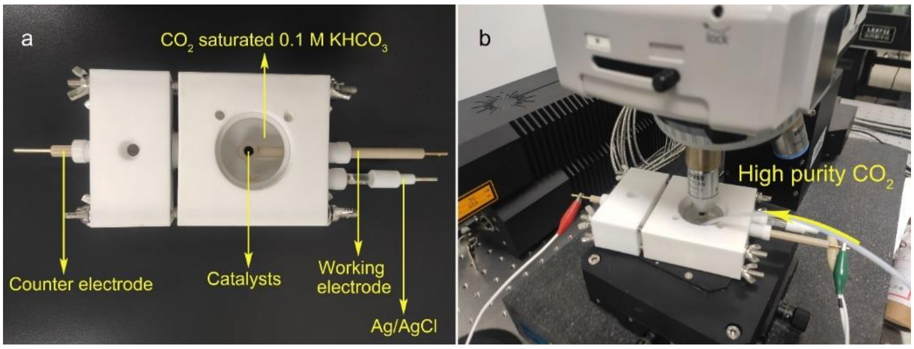

Figure S25. a) The electrochemical cell for in situ Raman spectroscopy measurement. b) The experimental set up for the in situ Raman spectroscopy measurement. High-purity CO_2 is continuingly introduced into the electrolyte and removed once the signal is acquired.

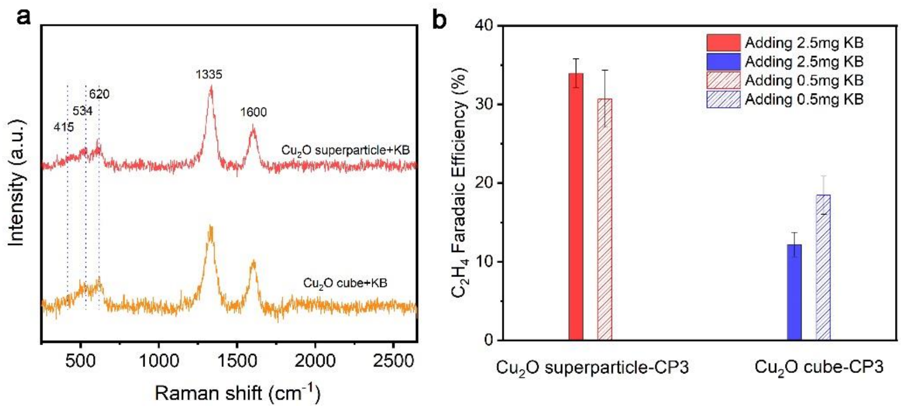

Figure S26. a) Raman spectra of the dried Cu_2O superparticle and Cu_2O cube ink. b) Comparison of C_2H_4 FE at −0.95 V (vs RHE) for adding different amount of Ketjen black carbon (KB) in the ink.

Similar to the FTIR characterization, too much KB in the ink will block the Raman signal of Cu_2O and intermediates. For this reason, addition of KB in the ink is reduced from 2.5 mg to 0.5 mg. Figure S22b shows that reducing KB to 0.5 mg in the catalyst ink does not lead to a significant difference on the FE of C_2H_4 at −0.95 V (vs RHE, within the potential range for in situ Raman spectroscopy characterization). As a result, in situ Raman spectroscopy characterization with 0.5 mg KB added can obtain reasonable data.

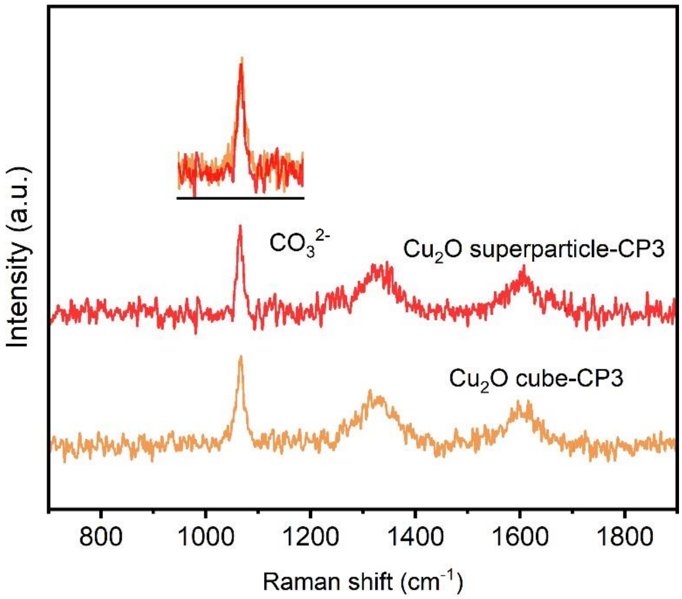

Figure S27. Raman spectra of Cu_2O superparticle-CP3 and Cu_2O cube-CP3 immersed in 1 M K_2CO_3 solution.

The SERS effect is typically generated on rough surfaces, especially at hot spots like tips, edges and gaps between two nanoparticles.[4] Cu_2O superparticle-CP3 is rich in defects and nano-gaps. Meanwhile, the surface of the Cu_2O cube becomes rough after pre-electroreduction. Both structures of the two catalysts can activate the SERS effects. Previous researches have revealed that the Raman intensity enhancement is dependent on the surface roughness.[5] Based on the results of underpotential deposition of lead (Pb-UPD), the electrochemical active surface areas (ECSA) of Cu_2O superparticle-CP3 and Cu_2O cube-CP3 are 1.227 cm^2 and 1.231 cm^2, respectively. The surface roughness factor is obtained by dividing ECSA by the geometric area of the used glass carbon electrode (0.196 cm^2). The results show that the roughness factors of Cu_2O superparticle-CP3 (6.26) and Cu_2O cube-CP3 (6.28) are very close, which should be the reason why the two catalysts have similar SERS effects.

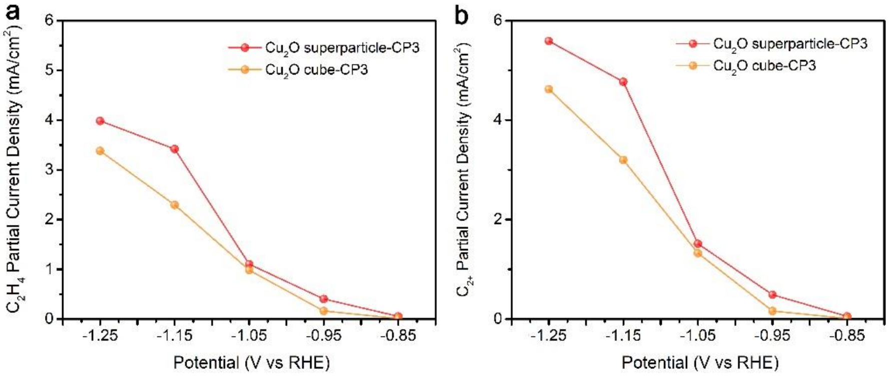

Figure S28. ECSA-normalized partial current densities of a) C_2H_4 and b) C_{2+} products toward Cu_2O superparticle-CP3 and Cu_2O cube-CP3. The current density (normalized by ECSA) at each potential is the average value of triplicate measurements.

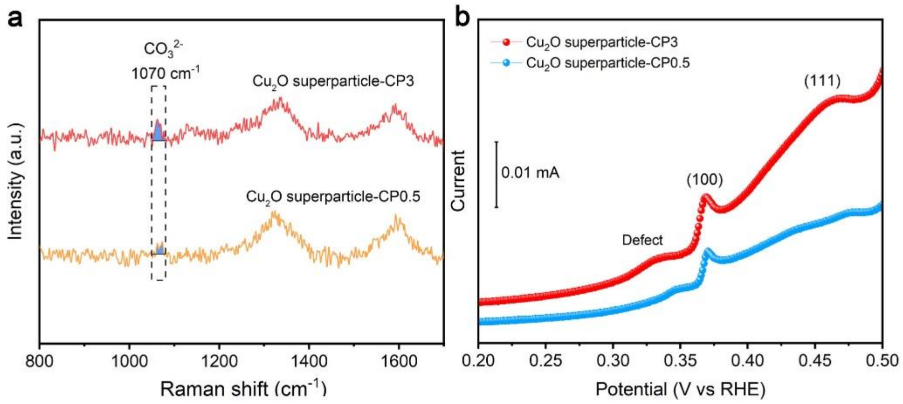

Figure S29. a) In situ Raman spectra of Cu_2O superparticle-CP3 and Cu_2O superparticle-CP0.5 at −0.95 V (vs RHE). b) OH^- electrosorption of Cu_2O superparticle-CP3 and Cu_2O cube-CP0.5.

Table S1. Performance comparison of the reported Cu-based electrocatalysts for CO_2 RR to C_2H_4 and C_{2+} products under comparable conditions in H-type cell.

Sample	Substrate	Electrolyte	E vs RHE	FE (C_2H_4)	FE (C_{2+})	Ref.
**Cu_2O superpaerticle-CP3**	Glass carbon	0.1 M $KHCO_3$	−1.15 V	53.2%	74.2%	**This work**
**Cu_2O cube-CP3**	Glass carbon	0.1 M $KHCO_3$	−1.15 V	40.1%	55.9%	
**Commercial Cu particles/C**	Glass carbon	0.1 M $KHCO_3$	−1.15 V	21%	28.5%	
**44 nm Cu cube**	Glassy carbon	0.1 M $KHCO_3$	−1.1 V	41.1%	50.1%	[6]
**Cu octahedra**	Glassy carbon	0.1 M $KHCO_3$	−1.15 V	~19%	~27%	[7]
**Cu star decahedron**	Glassy carbon	0.1 M $KHCO_3$	−0.993 V	52.4%	52.9%	[8]
**Cu mesocrystals**	Cu metal discs	0.1 M $KHCO_3$	−0.99 V	26.9%	27.2%	[9]
**100-cycle Cu**	Cu foil	0.25 M $KHCO_3$	−0.963 V	32%	60.5%	[10]
**1.7 µm Cu_2O fim**	Cu metal discs	0.1 M $KHCO_3$	−0.99 V	38.8%	47.9%	[11]
**Cu_2O cube/C**	Glassy carbon	0.5 M $KHCO_3$	−1.2 V	40%	N/A	[12]
**Cu_2O octahedra/C**	Glassy carbon	0.5 M $KHCO_3$	−1.1 V	45%	N/A	
**Fragmented Cu**	Glassy carbon	0.1 M $KHCO_3$	−1.1 V	57.3%	74%	[13]
**Cu@CuO NP/C**	Glassy carbon	0.1 M $KHCO_3$	−1.1 V	33.2%	44.5%	
**H-Cu nanowires**	Glassy carbon	0.1 M $KHCO_3$	−1.0 V	52.0%	71.9%	[14]
**A-Cu nanowires**	Glassy carbon	0.1 M $KHCO_3$	−1.0 V	57.7%	78.4%	
**Stepped surface Cu**	Glassy carbon	0.1 M $KHCO_3$	−1.0 V	69.8%	73.7%	[15]
**Branched CuO NPs**	Glassy carbon	0.1 M $KHCO_3$	−1.05 V	~70%	~70%	[16]
**Oxygen-bearing copper**	Cu foam	0.5 M $KHCO_3$	−0.95 V	45%	N/A	[17]
**MOF-derived Cu**	Glassy carbon	0.1 M $KHCO_3$	−1.4 V	45%	N/A	[18]
**EDTA-Cu**	Carbon paper	0.1 M $KHCO_3$	−0.82 V	50.1%	67.3%	[19]
**PANI-Cu nanoparticles**	Glassy carbon	0.1 M $KHCO_3$	−1.08 V	43.8%	77.4%	[20]

N/A: The data is no available.

Table S2. Fitting results of Cu K-edge EXAFS data.

Sample	Bond	CN	R (Å)	σ^2 (Å)	R-factor
**Cu foil**	Cu-Cu	12 (fixed)	2.54±0.01	0.00833	0.0088
**Cu_2O**	Cu-O	2 (fixed)	1.84±0.01	0.00288	0.0072
	Cu-Cu	12 (fixed)	3.05±0.03	0.02617	
**Cu_2O superparticle**	Cu-O	1.97±0.12	1.86±0.03	0.00187	0.011
	Cu-Cu	9.50±1.15	3.04±0.06	0.03070	
**Cu_2O cube**	Cu-O	2.00±0.07	1.85±0.02	0.00167	0.0034
	Cu-Cu	11.39±0.64	3.05±0.06	0.02902	
**Cu_2O superparticle-CP3 at −1.15 V (vs RHE)**	Cu-Cu	10.86±0.90	2.54±0.01	0.00839	0.0096
**Cu_2O cube-CP3 at −1.15 V (vs RHE)**	Cu-Cu	10.88±1.26	2.54±0.02	0.00829	0.018

CN, the coordination number; R, the bonding distance; σ^2, the Debye-Waller factor.

The Cu_2O superparticle and the Cu_2O cube have similar size of about 130 nm. However, from the EXAFS fitting results, we can clearly see that the Cu-Cu CN of Cu_2O superparticle (CN=9.50) is significantly lower than that of Cu_2O cube (CN=11.39), suggesting that the Cu_2O superparticle is comprised of smaller crystallites. Moreover, the Cu-O CN of Cu_2O superparticle (CN=1.97) is only slightly lower than that of Cu_2O cube (CN=2.00). This feature can be explained by the fact that the building blocks of Cu_2O superparticle are covered by PVP. PVP can establish coordination interaction with the Cu_2O surface through its carbonyl group.[21]

References

[1] T. Moeller, F. Scholten, T. Trung Ngo, I. Sinev, J. Timoshenko, X. Wang, Z. Jovanov, M. Gliech, B. Roldan Cuenya, A. Sofia Varela, P. Strasser, *Angew. Chem. Int. Ed.* **2020**, *59*, 17974.

[2] a) S. Popovic, M. Smiljanic, P. Jovanovic, J. Vavra, R. Buonsanti, N. Hodnik, *Angew. Chem. Int. Ed.* **2020**, *59*, 14736; b) F. Li, X. V. Medvedeva, J. J. Medvedev, E. Khairullina, H. Engelhardt, S. Chandrasekar, Y. Guo, J. Jin, A. Lee, H. Therien-Aubin, A. Ahmed, Y. Pang, A. Klinkova, *Nat. Catal.* **2021**, *4*, 479.

[3] a) K. Manthiram, Y. Surendranath, A. P. Alivisatos, *J. Am. Chem. Soc.* **2014**, *136*, 7237; b) W. T. Osowiecki, J. J. Nussbaum, G. A. Kamat, G. Katsoukis, M. Ledendecker, H. Frei, A. T. Bell, A. P. Alivisatos, *ACS Appl. Energy Mater.* **2019**, *2*, 7744.

[4] Y. Zhao, X. Chang, A. S. Malkani, X. Yang, L. Thompson, F. Jiao, B. Xu, *J. Am. Chem. Soc.* **2020**, *142*, 9735.

[5] a) Z. Q. Tian, B. Ren, D. Y. Wu, *J. Phys. Chem. B* **2002**, *106*, 9463; b) Y. Zhao, X. Liu, D. Y. Lei, Y. Chai, *Nanoscale* **2014**, *6*, 1311.

[6] A. Loiudice, P. Lobaccaro, E. A. Kamali, T. Thao, B. H. Huang, J. W. Ager, R. Buonsanti, *Angew. Chem. Int. Ed.* **2016**, *55*, 5789.

[7] N. T. Suen, Z. R. Kong, C. S. Hsu, H. C. Chen, C. W. Tung, Y. R. Lu, C. L. Dong, C. C. Shen, J. C. Chung, H. M. Chen, *ACS Catal.* **2019**, *9*, 5217.

[8] C. Choi, T. Cheng, M. F. Espinosa, H. Fei, X. Duan, W. A. Goddard, III, Y. Huang, *Adv. Mater.* **2019**, *31*, 1805405.

[9] C. S. Chen, A. D. Handoko, J. H. Wan, L. Ma, D. Ren, B. S. Yeo, *Catal. Sci. Technol.* **2015**, *5*, 161.

[10] K. Jiang, R. B. Sandberg, A. J. Akey, X. Liu, D. C. Bell, J. K. Norskov, K. Chan, H. Wang, *Nat. Cata.* **2018**, *1*, 111.

[11] D. Ren, Y. Deng, A. D. Handoko, C. S. Chen, S. Malkhandi, B. S. Yeo, *ACS Catal.* **2015**, *5*, 2814.

[12] Y. Gao, Q. Wu, X. Liang, Z. Wang, Z. Zheng, P. Wang, Y. Liu, Y. Dai, M.-H. Whangbo, B. Huang, *Adv. Sci.* **2020**, *7*, 1902820.

[13] H. Jung, S. Y. Lee, C. W. Lee, M. K. Cho, D. H. Won, C. Kim, H.-S. Oh, B. K. Min, Y. J. Hwang, *J. Am. Chem. Soc.* **2019**, *141*, 4624.

[14] Z. Lyu, S. Zhu, M. Xie, Y. Zhang, Z. Chen, R. Chen, M. Tian, M. Chi, M. Shao, Y. Xia, *Angew. Chem. Int. Ed.* **2020**, *59*, 2.

[15] C. Choi, S. Kwon, T. Cheng, M. Xu, P. Tieu, C. Lee, J. Cai, H. M. Lee, X. Pan, X. Duan, W. A. Goddard, III, Y. Huang, *Nat. Cata.* **2020**, *3*, 804.

[16] J. Kim, W. Choi, J. W. Park, C. Kim, M. Kim, H. Song, *J. Am. Chem. Soc.* **2019**, *141*, 6986.

[17] W. Zhang, C. Huang, Q. Xiao, L. Yu, L. Shuai, P. An, J. Zhang, M. Qiu, Z. Ren, Y. Yu, *J. Am. Chem. Soc.* **2020**, *142*, 11417.

[18] F. Yang, A. Chen, P. L. Deng, Y. Zhou, Z. Shahid, H. Liu, B. Y. Xia, *Chem. Sci.* **2019**, *10*, 7975.

[19] J. Liu, J. Fu, Y. Zhou, W. Zhu, L.-P. Jiang, Y. Lin, *Nano Lett.* **2020**, *20*, 4823.

[20] X. Wei, Z. Yin, K. Lyu, Z. Li, J. Gong, G. Wang, L. Xiao, J. Lu, L. Zhuang, *ACS Catal.* **2020**, *10*, 4103.

[21] A. Nemamcha, J. L. Rehspringer, D. Khatmi, *J. Phys. Chem. B.* **2006**, *110*, 383.
